# Cell-membrane-coated nanoparticles for the fight against pathogenic bacteria, toxins, and inflammatory cytokines associated with sepsis

**DOI:** 10.7150/thno.81520

**Published:** 2023-05-21

**Authors:** Xiaoyi Wang, Zongping Xia, Huaili Wang, Dao Wang, Tongwen Sun, Eamran Hossain, Xin Pang, Yufeng Liu

**Affiliations:** 1 Henan Key Laboratory of Pediatric Hematology and Oncology Medicine, Department of Pediatrics, The First Affiliated Hospital of Zhengzhou University, Zhengzhou 450052, China.; 2 Department of Clinical Systems Biology Laboratories, The First Affiliated Hospital of Zhengzhou University, Zhengzhou 450052, China.; 3 Department of Integrated ICU, The First Affiliated Hospital of Zhengzhou University, Zhengzhou 450052, China.; 4 School of Pharmacy, Henan University of Chinese Medicine, Zhengzhou 450046, China.

**Keywords:** cell membrane-coated nanoparticle, bacterial sepsis, multifunctionality, toxin, inflammatory cytokine

## Abstract

Sepsis is the main cause of death in patients suffering from serious illness. Yet, there is still no specific treatment for sepsis, and management relies on infection control. Cell membrane-coated nanoparticles (MNPs) are a new class of biomimetic nanoparticles based on covering the surface of synthetic nanoparticles (NPs) with natural cell membranes. They retain the physicochemical properties of synthetic nanomaterials and inherit the specific properties of cellular membranes, showing excellent biological compatibility, enhanced biointerfacing capabilities, capacity to hold cellular functions and characteristics, immunological escape, and longer half-life when in circulation. Additionally, they prevent the decomposition of the encapsulated drug and active targeting. Over the years, studies on MNPs have multiplied and a breakthrough has been achieved for cancer therapy. Nevertheless, the use of “bio”-related approaches is still rare for treating sepsis. Herein, we discussed current state-of-the-art on MNPs for the treatment of bacterial sepsis by combining the pathophysiology and therapeutic benefits of sepsis, i.e., pathogenic bacteria, bacteria-producing toxins, and inflammatory cytokines produced in the dysregulated inflammatory response associated with sepsis.

## Introduction

Sepsis is a serious medical condition that occurs when the body has an overwhelming immune response to an infection. It can lead to tissue damage, organ failure, and even death 1. It is estimated to cause 20.7 million cases per year and up to 5.3 million deaths per year 2. Clinically, Sepsis causes a range of symptoms, such as a high temperature, an increased heart rate, and rapid breathing, which can lead to serious organ damage and even death, which may resemble coronavirus disease 2019. Over a hundred clinical trials have been conducted for treatments related to sepsis; however, no Food and Drug Administration-approved treatment options for sepsis have been developed 3. Therefore, exploring innovative and effective therapies to improve the prognosis of individuals suffering from sepsis is of utmost importance.

Over the years, NPs have become increasingly prevalent in medical research due to their distinct characteristics, such as functional surfaces, small size, and pharmacokinetic/biodistribution profiles 4, 5. Several methods of NP drug delivery have been explored as potential ways to deliver anti-septic treatments 6. Nevertheless, once in the body, NPs are recognized as foreign substances, which are then easily eliminated by a passive immune clearance 7. To address these shortcomings, surface modification with the hydrophilic polyethylene glycol (PEG) domain, also known as PEGylation, has been proposed as a functionalisation required to optimize the NP performance in biological systems due to the stealth effect of PEG, which effectively shields the negative effects of the cationic domains on the surface of NP, preventing non-specific protein adsorption and improving the protease stability and biocompatibility of NPs 8, 9. An overview of treatment strategies directed at pathogenic bacteria in sepsis is summarized in **Table S1**.

In terms of eliminating pathogenic bacteria, an antibiotic is regarded as the most efficient drug against sepsis 10. However, the overutilization of antibiotics leads to the rapid appearance of multidrug resistant (MDR) “superbugs”. Sepsis treatment strategies based on antibiotics focus on eliminating bacteria rather than toxins and inflammatory cytokines. It is well known that removing them could be used as an adjuvant approach to antibiotics and supportive care to bring additional benefits to the survival of sepsis 11-13. Some treatment strategies aimed at toxins and inflammatory cytokines in sepsis are listed in **Table S2** and **Table S3**, respectively. In the beginning, researchers dedicated much efforts to structure-based neutralization strategies for removing inflammatory mediators by using cytokine-neutralizing antibodies, such as tumor necrosis factor (TNF)-α antibody, interferon (IFN)-γ antibody, IL-1 antibody, and IL-6 antibody *et al*.; still, none of the anti-inflammation agents had any tangible outcomes 14. Differences in structural motifs and the wide array of toxins released by various bacterial genera, species and strains, make it difficult to target a treatment that effectively neutralizes narrow-spectrum toxins 15. In addition, multiple toxins and inflammatory mediators contribute dynamically to systemic inflammation in sepsis. Still, attempting to disrupt a single mediator may not be enough to stop the intricate inflammatory reaction 16. To overcome these challenges in structure-based strategies, blood purification strategies have evolved as a novel adjunctive therapy that reduces a wide variety of toxins and inflammatory agents from the body in a non-specific approach, leading to increased survival in sepsis 17, 18. However, the above treatment strategies aim to target only one pathogenic factor: bacteria, toxins, or inflammatory cytokines. Also, the roots and routes of sepsis are certainly multifactorial and may influence and interact with one another 19, 20.

In recent years, a multi-target strategy based on NPs for sepsis treatment has been proposed and has become a crucial research focus. However, this process is extremely challenging 21. To address the challenges of NPs, researchers have been exploring biomimetic nanoparticles, which draw inspiration from nature to obtain desired properties. This concept of “learning from nature” is helping to advance nanoparticle technology 22. Imitating cells, which are one of the most essential components of biology, has been used in biomimetics 23. A captivating potential has been identified through the usage of a top-down fabrication approach to coat natural cellular membranes onto synthetic NP. It is well known that the cell membrane has many essential functions that define the biological identity and behavior 24. The first function is active targeting, which is mediated by the interaction of membrane proteins with specific molecules expressed by tissues. The second function is biocompatibility, self-recognition, which avoids immune clearance by phagocytosis and decreases the retention effect by the reticuloendothelial system (RES), allowing a prolonged plasmatic half-life. The last function is “decoy”, which is similar to sponges with the capacity of “soaking up” harmful toxins, pathogens, or pro-inflammatory cytokines for neutralization 25. In the nanoformulation fabricated by camouflaging NP with the cell membrane, the exterior plasma membrane coating inherits the above functions. The interior of the NP acts as a stabilizing agent for the exterior membrane shell, and is used to facilitate medication delivery. Therefore, MNPs have the functions of both NPs and cell membranes, including drug delivery, targeting, biocompatibility, and neutralizing toxins and/or inflammatory cytokines, which enables MNPs to fight against muti-targets in a single treatment 26.

**Figure 1:**
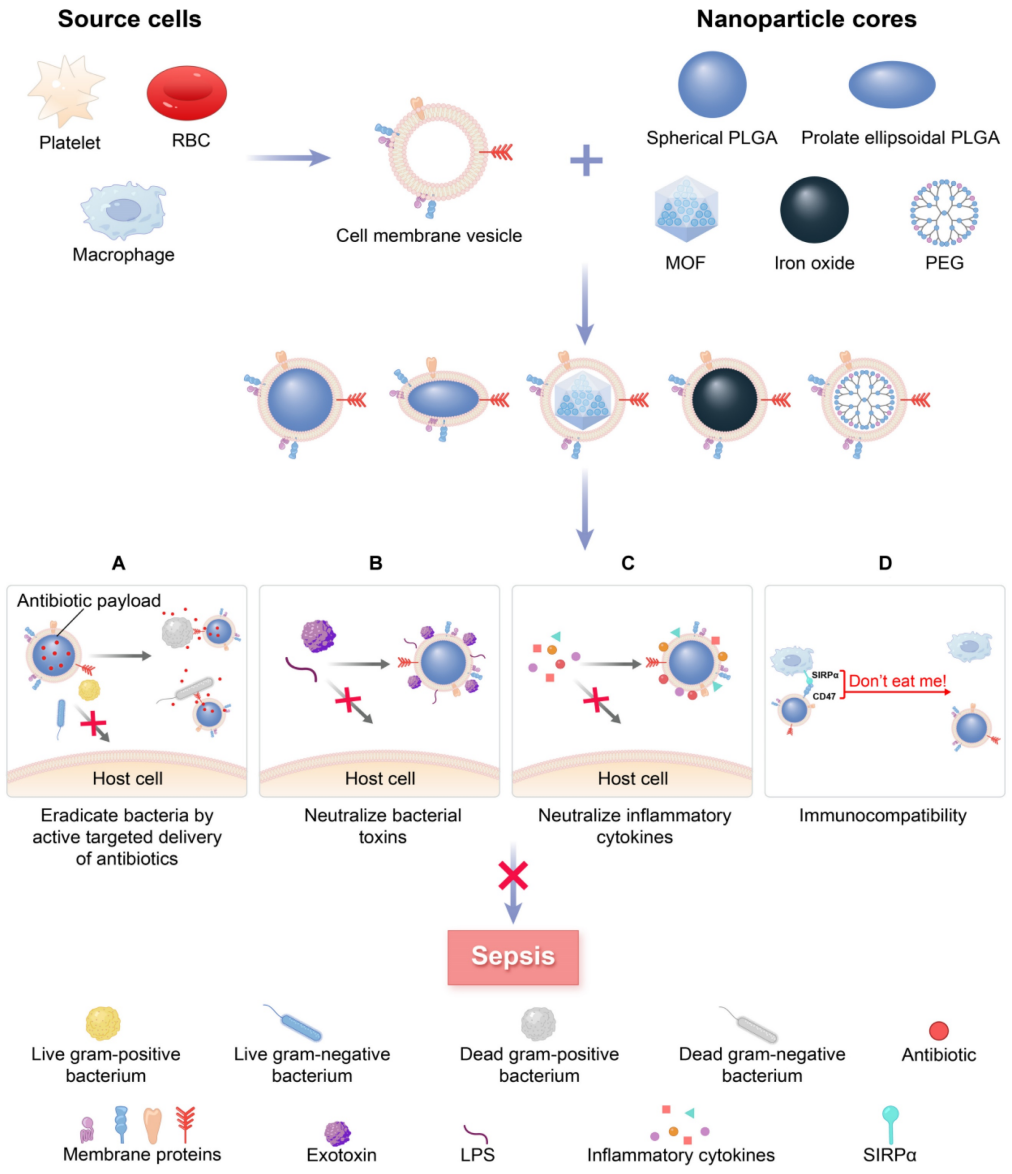
** A schematic summary of the use of cell membrane-coated NPs (MNPs) for sepsis therapy.** MNPs are made by cloaking plasma membranes derived from platelets (PLs), red blood cells (RBCs), and macrophages on synthetic cores, including NPs of spherical and prolate ellipsoidal PLGA, MOF, iron oxide, and PEG. The resulting MNPs are multifunctional, allowing MNPs to eradicate bacteria by active targeted delivery of antibiotics (**A**), countering a wide range of toxins with non-specific neutralization (**B**), controlling inflammatory agents (**C**), and preventing the immune system clearing (**D**) to treat sepsis.

Specifically, MNPs can eliminate bacteria by accurately administering antibiotics in the treatment of bacterial sepsis (**Figure 1A**). In contrast to traditional NPs, the new biomimetic NPs enhance the effectiveness of antibiotics through improved biodistribution and bioavailability, thereby reducing the need for excessive antibiotic use and diminishing the development of antibiotic resistance. This allows for a longer lifespan of newly developed antibiotics 27. MNPs have the potential to bind and neutralize a broad array of toxins and inflammatory agents that may be found in sepsis. These particles are capable of non-specifically adsorbing a variety of these substances 28, 29 (**Figure 1B-C**). Importantly, the biocompatibility of MNPs prolongs the blood circulation of NPs by reducing clearance by the immune system (**Figure 1D**). Overall, MNPs achieve a synergistic effect for sepsis treatment by fighting against muti-targets, and thus therapeutic benefits can be maximized.

There have been significant research interests in the MNPs over the past decade. Thus far, researchers have summarized nanotechnology-based therapeutic strategies toward sepsis while only involving MNPs without making a more comprehensive summary. Considering that several emerging MNP-based treatments for sepsis have recently been reported with promising results, a pertinent review is needed. In this review, we discussed the latest developments in the use of MNPs for treating bacterial sepsis by combining the pathophysiology and therapeutic benefits of sepsis.

## 1. The pathogenesis of bacterial sepsis focused on bacteria, toxins, and dysregulated inflammation

### 1.1 Pathogenic bacteria

**Figure 2:**
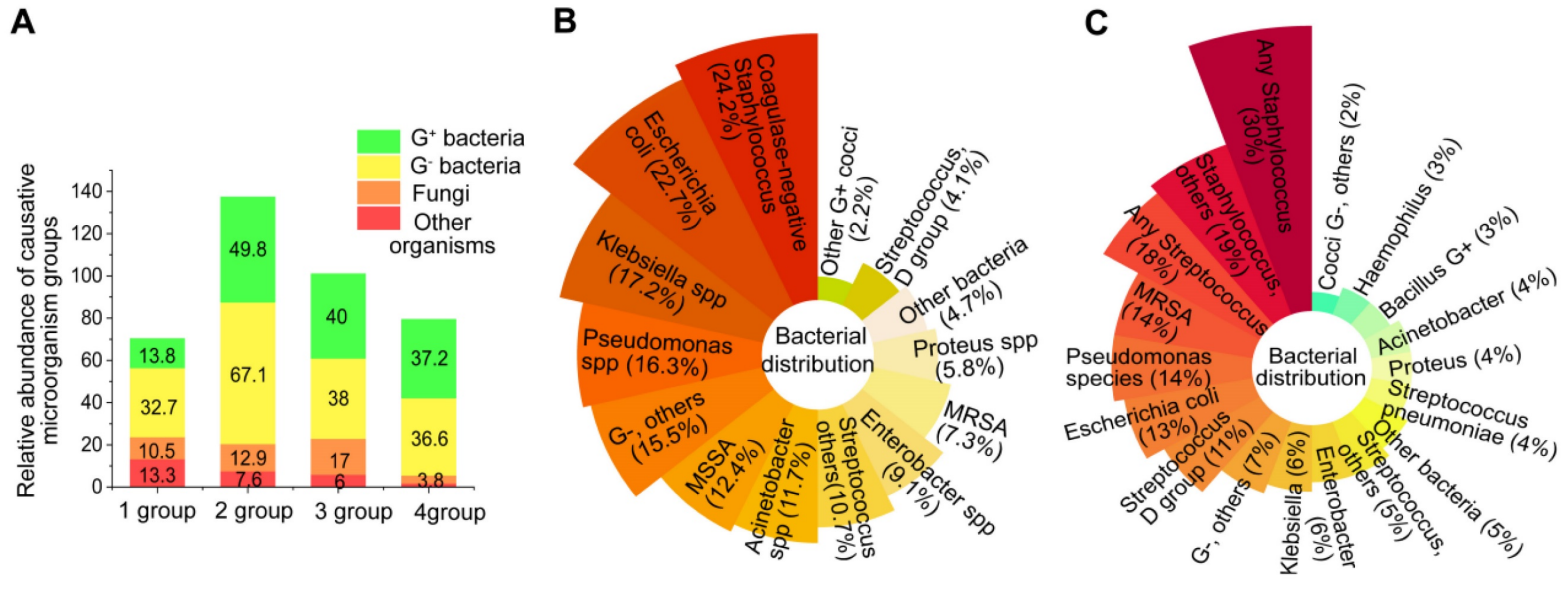
**The abundance of microorganisms that cause sepsis**. (**A**) The abundance of etiological agents in individuals with sepsis among four demographic groups. (**B**) Distribution of bacteria in individuals from the Intensive Care Over Nations audit. (**C**) The distribution of bacteria in septic patients from European intensive care units.

**Figure 3:**
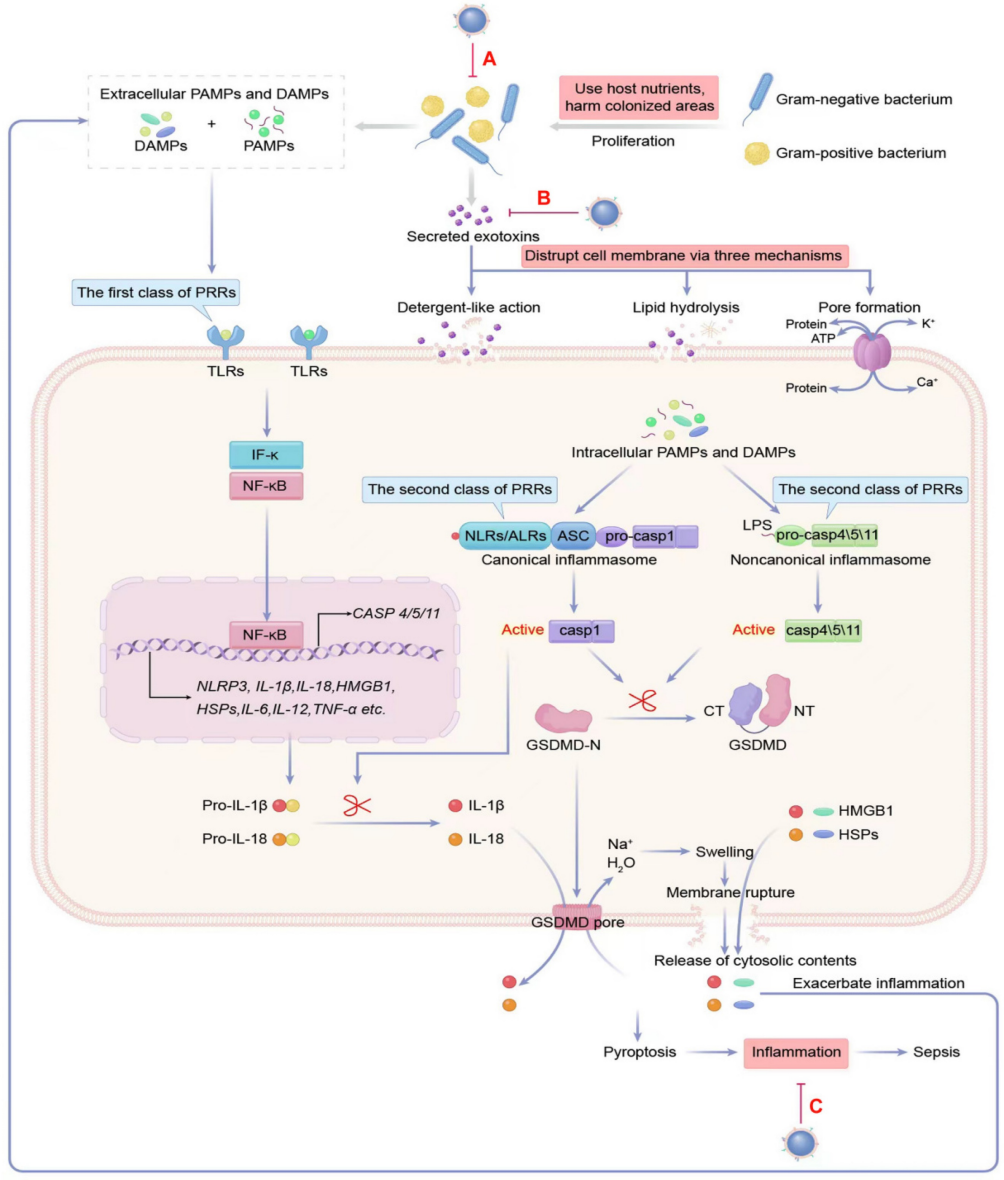
**The pathogenesis of bacterial sepsis is caused by bacteria, toxins, and inflammation.** The damage inflicted on the septic host is attributable to the growth of bacteria, the toxins produced by bacteria that disrupt the cell membrane, and the host responses to PAMPs and DAMPs by triggering dysregulated inflammation. Therefore, all three are considered potential therapeutic targets of MNPs for sepsis (**A-C**). DAMPs: danger-associated molecular pattern molecules; HMGB1: high-mobility group box 1; HSP: heat-shock protein; IFN: interferon; IL: interleukin; LPS: lipopolysaccharide; NLRs: NOD-like receptors; PAMPs: pathogen-associated molecular pattern molecules; PRRs: pattern recognition receptors; TLRs: toll-like receptors; TNF: tumor necrosis factor.

**Figure 4:**
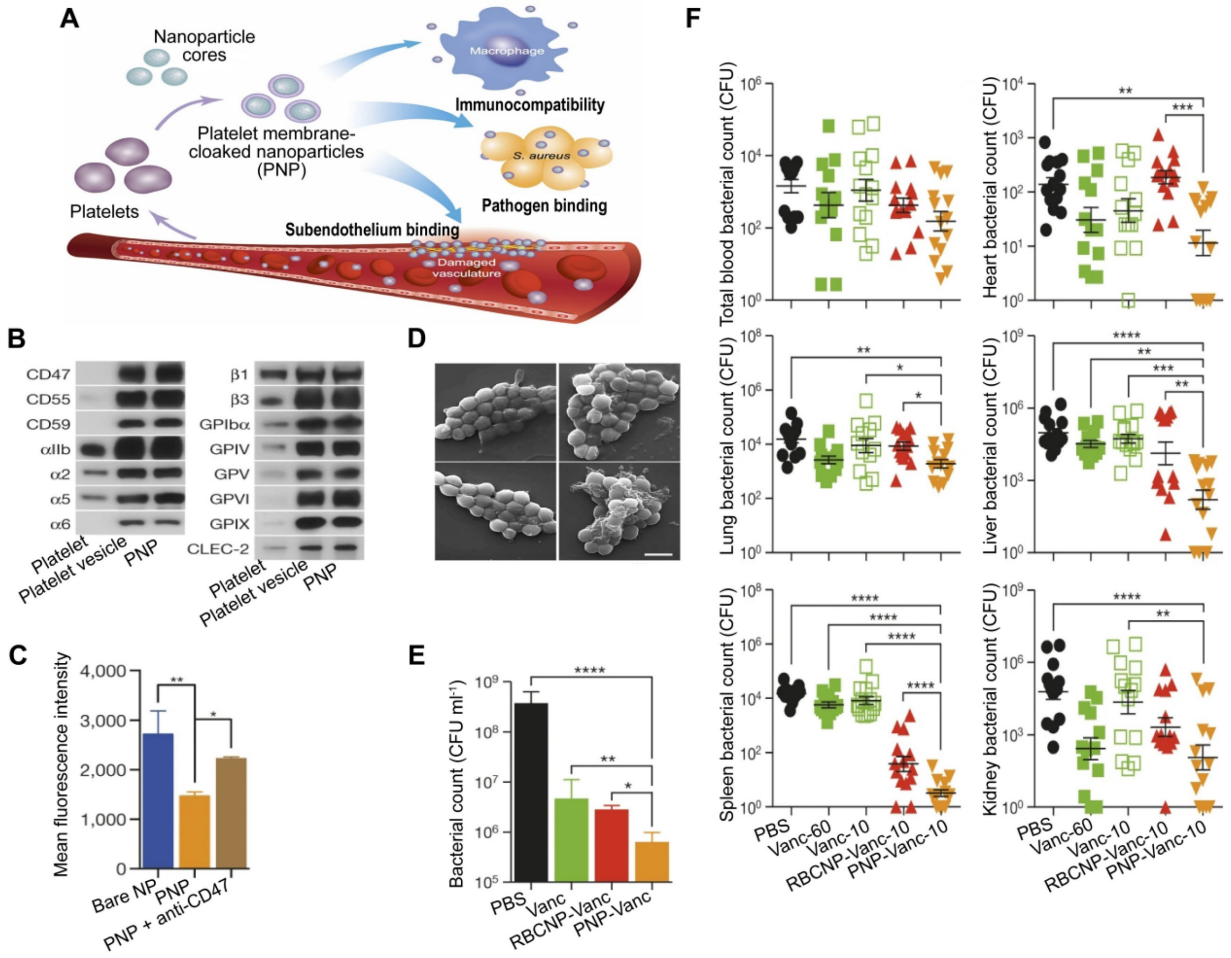
** PNPs for sepsis treatment based on actively targeting and killing bacteria.** (**A**) Schematic illustration of the constitution and properties of PNPs. (**B**) Qualitative analysis of representative PL membrane proteins by Western blotting. (**C**) Flow cytometric analysis of nanoparticles that have been phagocytized by human THP-1 cells. (**D**) Visualization of MRSA252 bacteria using scanning tunnel microscopy after incubation with PBS (top left), uncoated nanoparticles (top right), RBCNPs (bottom left), and PNPs (bottom right). Scale bar = 1 μm. (**E**) *In vitro* antimicrobial efficacy of Van in different forms. (**F**) *In vivo* antimicrobial efficacy of Van evaluated by quantifying bacterial counts in mice's blood, heart, lung, liver, spleen, and kidney, which were systemically infected with MRSA252 and treated with Van in different forms and doses for 3 days. ^*^P ≤ 0.05, ^**^P ≤ 0.01, ^***^P ≤ 0.001, ^****^P ≤ 0.0001. Adapted with permission from 76, copyright Year 2015 Springer Nature.

Sepsis is a potentially life-threatening condition resulting from an infection with either gram-negative or gram-positive bacteria 30, 31. In a study of Chinese intensive care unit patients with sepsis, gram-negative bacteria were found to be the most common, accounting for 32.7% of cases, followed by gram-positive bacteria which accounted for 13.8% 32 (**Figure 2A, 1 group**). Sakr *et al.* 33 observed similar data that evaluated global records from the Intensive Care over Nations audit (**Figure 2A, 2 group**). Conversely, the population studies by Vincent *et al.* 34 and Opal *et al.* 35 showed approximately the same number of gram-positive and gram-negative bacterial infections. (**Figure 2A, 3 and 4 group**).

In relation to particular species, the most common bacteria responsible for sepsis are typically *S. aureus*, *Streptococcus*, *E. coli, Pseudomonas species,* and *Klebsiella spp* (**Figure 2B-C**). However, several bacteria, including *Acinetobacter*, *Enterobacter*, *S. aureus*,* Streptococcus pneumoniae* and *Haemophilus*, appear on the list of deadly drug resistant bacteria recently published by the World Health Organisation 36. The effects of pathogenic bacteria on the pathogenesis of bacterial sepsis are shown in **Figure 3**.

### 1.2 Toxins

Apart from bacteria causing disease, bacteria that produce toxins are also hugely important in the development of bacterial sepsis 37. The interaction of toxins with the cell membrane is essential to inflict virulence. Generally, toxins can target specific biomolecules on membrane surfaces, such as proteins, lipid derivatives, and cholesterol. Alternatively, they can be bound via nonspecific electrostatic interactions 38. Based on location distribution, bacterial toxins are divided into exotoxins and endotoxins 39.

Exotoxins are secreted by bacteria into the environment and are classified into three types according to the mode of action: superantigens (Type I), agents that disrupt cell membranes (Type II), and A-B toxins (Type III) 40. Among them, membrane-disrupting toxins damage cell membranes using one of the following mechanisms (**Figure 3**): (1) detergent-like action: solubilizing membranes in a detergent-like fashion, e.g., δ-Toxins from staphylococcal species; (2) hydrolyzing lipids by enzymatic phospholipase activity and causing the breakdown of membranes by targeting ester bonds, e.g., β-hemolysin from Staphylococcus aureus; (3) pore formation: monomer pore-forming toxins (PFTs) bind to target cells to favor oligomerization and eventually create hydrophilic “holes” that provide a channel for solutes (water, ions, or other biomolecules) across diverse target membranes.

A large number of membrane-damaging proteins are part of the PFTs. These proteins account for roughly 30% of all toxins found in bacteria that cause illness 41. The formation of membrane pores is not only a method used to immediately lyse the cell of interest, but it is also a way to enable the penetration of epithelial barriers and evasion of the body's immune response, providing a location for pathogenic bacteria to survive 42. Cases of sepsis involving PFTs involve β-Hemolysin/Cytolysin from Group B Streptococcus 43, the colicin family from *E. coli* (colicin E1, colicin Ia, colicin A and colicin N), cytolysin A family (cytolysin A from *E. coli, Salmonella enterica* and* Shigella flexneri*), enterotoxin from *Bacillus cereus*, and the hemolysin family from *S. aureus* (α-hemolysin (Hla), γ-haemolysin AB (HlgAB), HlgCB, and leukocidin AB) 44.

Endotoxins become toxic to host cells when released into the bloodstream as a result of bacterial cell death and lysis. Most endotoxins belong to lipids, while exotoxins are proteins. The outer membrane of most gram-negative bacteria is composed of lipopolysaccharide (LPS), which makes up around 75% of the membrane and is considered to be the classic endotoxin. LPS resulting from uncontrolled infection induces macrophages to release a high level of cytokines; therefore, it is a crucially important microbial toxin in the pathogenesis of sepsis 45, 46. Evidence is growing that the systemic dissemination of endotoxin from sources of infection, rather than bacteremia itself, is a critical factor in the development of serious immune disturbances 47.

### 1.3 Dysregulated inflammatory response

In the initial stage of sepsis, the body's immune system is activated and triggers an intense inflammatory reaction, referred to as a “cytokine storm”. This response is caused by molecules known as pathogen-associated molecular pattern molecules (PAMPs) which are produced by bacteria and their toxins. Also, danger-associated molecular pattern molecules (DAMPs) released upon host cell death can trigger inflammation 48-50. However, over time, a prolonged or severe low inflammatory state results in the failure of the immune effector, that is, an immunosuppression state, which implicates that the immune system can be severely compromised, including a depletion of T cells, the most affected cell type. Furthermore, there are associated T cell effector functions, T cell exhaustion, and impaired antigen presentation that can lead to viral reactivation, secondary infection, and long-term mortality one year after sepsis 51-53.

The host's immune system makes use of pattern recognition receptors (PRRs) on sentinel cells to recognize PAMPs such as bacterial cell wall components, microbial nucleic acids, and bacterial secretion systems, as well as DAMPs like high-mobility group box 1 (HMGB1), uric acid crystals, the heat-shock protein (hsp) 70 and 90, and ATP 54, 55. PRRs are divided into two types according to their subcellular location: the first type of PRRs are Toll-like receptors (TLRs) and C-type lectin-like receptors, which detect pathogens either the cell surface or in the cytosolic organelles of cells and initiate the inflammatory response 56. Some transmembrane PRR and corresponding PAMPs and DAMPs participating in sepsis-associated inflammation are shown in **Table S4** 57-59.

Once inside the cytosol, invading pathogens are detected by C-type lectin-like receptors, which are housed within the cytoplasm. These cytoplasmic sensors include NOD-like receptors (NLRs), AIM-2-like receptors (ALRs), and cytosolic nucleoside sensors 60. The combination of NLRs and ALRs with their specific ligands creates a cytosolic inflammatory complex known as the canonical inflammasomes, which is responsible for activating caspase-1, a pro-inflammatory enzyme. The noncanonical inflammasome can detect lipopolysaccharide (LPS) in the cytosol and activate human caspase-4/5 and murine caspase-11 61-63. The activation of caspase-1/4/5/11 in canonical and noncanonical inflammasomes triggers a swift and inflammatory form of cell death, called pyroptosis. This leads to (1) inflammation by forming gasdermin D pores in the plasma membrane, which causes the cell to swell and the membrane to break, leading to the release of large amounts of cytosolic material. These contents are then recognized as danger signals, namely DAMPs, and propagate inflammatory responses through various mechanisms 64. (2) The release of interleukin (IL)-1β and IL-18 through gasdermin D pores can cause strong pro-inflammatory activity, leading to vasodilation and extravasation of immune response cells, generation of IL-17-producing helper T cells (Th17 response), and production of interferon-g by natural killer and Th1 cells 65, 66. Some cytoplasmic PRRs as well as corresponding PAMPs and DAMPs engaged in the inflammation, are summarized in **Table S5** 67, 68.

Numerous studies have identified that the inflammation in sepsis is caused primarily by the overactivation of TLRs 69 and the NLRs 70-72, which impair host defense against pathogens and have important roles in the exaggerated systemic inflammation and inflammatory organ damage during sepsis. Remarkably, recent evidence showed that sepsis cytokine storms are the result of synergistic interactions between TLR and NLRs 73.

## 2. Multifunctional MNPs platform may treat bacterial sepsis by targeting pathogens, toxins, and inflammatory cytokines

Multifunctional MNPs can be used to treat sepsis by simultaneously targeting pathogenic bacteria (**Figure 3A**), toxins (**Figure 3B**), and inflammatory cytokines (**Figure 3C**). In the last ten years, cell membranes from RBCs, leukocytes, PLs, and bacterial cells have been coated on NPs, enabling them to circulatory in the bloodstream for extended periods, achieve targeted drug delivery, or neutralize toxins and inflammatory cytokines. Based on the classification of cell membrane sources, the following section offers a systematic overview of MNPs for treating bacterial sepsis against bacteria, toxins, and inflammatory cytokines.

### 2.1 Platelet-membrane coated nanoparticles (PNPs) are being explored as a potential treatment for bacterial sepsis through actively targeting and destroying

PLs are important for the body's immune response to infection, and their involvement in the inflammation and coagulation issues that can cause harm to organs in the event of sepsis is well established 74. Additionally, PLs can interact with bacteria and bacteria-secreted toxins 75, which gives them the capacity for active targeting and detoxification. A type of NP made of poly(lactic-co-glycolic acid) (PLGA) that had a coating of plasma membranes derived from human PLs 76, named PNPs, has been created, which possess PL-mimicking properties, including good immunocompatibility, pathogen adhesion, and subendothelium binding (**Figure 4A**). PNPs express PL membrane proteins, including immunomodulatory proteins (cluster of differentiation (CD 59, CD 55, and CD 47), integrin factors (αIIb, α2, α5, α6, β1, and β3), and additional transmembrane proteins (GPIbα, GPIV, GPV, GPVI, GPIX, and CLEC-2) (**Figure 4B**), which reduce the internalization of particles by differentiated human THP-1 macrophage-like cells in a CD47-specific manner (**Figure 4C**). Compared to bare NPs in particle adhesion study, PNPs prefer to bind to MRSA252, a particular strain of Methicillin-resistant Staphylococcus aureus (MRSA) that expresses adherin with a high concentration of serine to attach to PLs (**Figure 4D**). Furthermore, the effectiveness of the PNPs was evaluated with vancomycin (Van)-loaded formulations, known as PNP-Van. The tests revealed a statistically significant reduction in the number of MRSA252 *in vitro* (**Figure 4E**) and *in vivo* (**Figure 4F**). Additionally, compared to Van given in a free form at six times the dosage, PNP-Van showed significantly more effective antimicrobial properties in the liver and spleen, with similar efficacy in the blood, heart, lung, and kidney.

In terms of the sepsis model, there were very few studies about MNPs taken as antibiotic carriers, whereas in other models of bacterial infection, such as the MRSA wound infection model 77-79 and the MRSA pulmonary inflammation model 80, researchers have demonstrated that the novel delivery system possesses excellent protective effects. Owing to the similarity of the above bacterial infection models with the sepsis model, such as pathogenic bacteria, toxins, and inflammation, it was further suggested that bioinspired membrane-coated nanoplatforms have enormous advantages in treating refractory sepsis.

### 2.2 NPs that are coated with RBC membranes (RBC NPs) utilized as treatment for bacterial sepsis by neutralizing multiple kinds of bacterial toxins and/or killing bacteria

**Figure 5:**
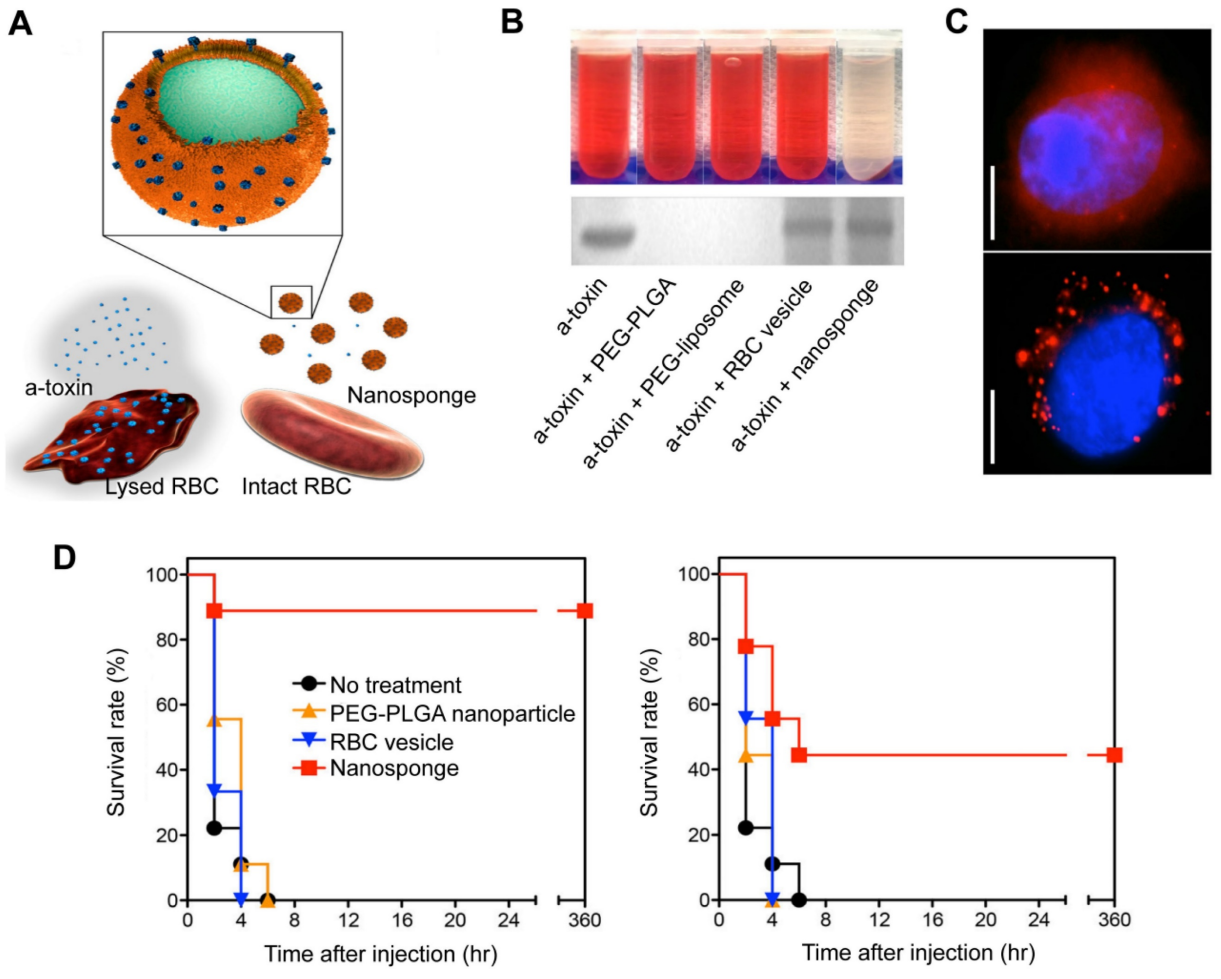
**RBC-NPs for the treatment of sepsis based on a detoxification effect.** (**A**) Schematic illustration of nanosponge neutralizing PFTs. (**B**) After mixing liposomes, RBC membrane vesicles, PBS, PLGA NPs, and nanosponges with α-toxin, the hemolytic effect was observed after centrifugation of the RBCs (top). The absorption of the α-toxin was evidenced by SDS-PAGE after filtration of the respective mixtures through a column (bottom). (**C**) Fluorescence images of red-colored vesicles from RBC membrane (top) and nanosponges (bottom) engulfed by human umbilical vein endothelial cells. Scale bar = 5 μm. (**D**) Survival rates of mice two minutes prior to or after the injection of the toxin. Adapted with permission from 82, copyright Year 2013 Springer Nature.

RBCs are naturally occurring, long-circulating carriers that can remain viable in human bodies for up to 120 days. The “marker of self” CD47 receptor on the surface of RBCs binds to the signal regulatory protein alpha receptor on macrophages, preventing phagocytosis and allowing RBC-NPs to remain in circulation for extended periods of time 81. In addition to this, RBC membranes can intercept and neutralize bacterial toxins because they have a natural affinity for toxins. In 2013, Hu *et al.* showed a detoxification effect of RBC membrane-coated PLGA NPs (denoted as nanosponge) (**Figure 5A**) in PFTs induced sepsis model 82. RBC lysis tests showed that, unlike the other samples, the nanosponge sample exhibited a clear supernatant, suggesting that nanosponges neutralize the cytotoxicity of α-toxin (**Figure 5B, top**). Furthermore, the SDS-PAGE results demonstrated that the nanosponges and the RBC membrane vesicles could effectively retain large amounts of α-toxin compared to the PLGA nanoparticle and liposome (**Figure 5B, bottom**). This suggests that RBC membrane vesicles can sponge α-toxin; yet, they do not diminish its hemolytic action. These findings underscore the importance of the polymeric cores in the nanosponges. Concretely, the observed hemolysis results were justified by fluorescence microscopy (**Figure 5C**). It was hypothesized that RBC membrane vesicles with α-toxin bound to them are expected to merge with RBCs, thus enabling the toxin to cause hemolysis. On the other hand, nanosponges can capture and keep toxins away from other RBC membranes, showing the importance of their polymeric core. Additionally, *in vivo* suggested that nanosponges can enhance the chances of survival for mice that have been exposed to a toxin (**Figure 5D**). Significantly, no further deaths occurred after 6 h in the nanosponge treatment groups, indicating that the absorbed toxins were cleared and not just delayed.

RBC-NPs have been used to neutralize and absorb a wide range of hemolytic toxins, no matter their molecular structure. In addition, these nanoparticles have been demonstrated to be effective against other types of toxins that target the membrane, such as melittin (a peptide found in bee venom that disrupts the membrane), α-hemolysin (from MRSA), and listeriolysin O (from Listeria monocytogenes) 83, streptolysin O (a toxin produced by Group A Streptococcus) 84, 85, the whole secreted proteins (wSP, MRSA) 86, and β-hemolysin/cytolysin (Group B *Streptococcus*) 87, consolidating this decoy strategy as versatile in many pathological contexts regardless of their molecular structure.

**Figure 6:**
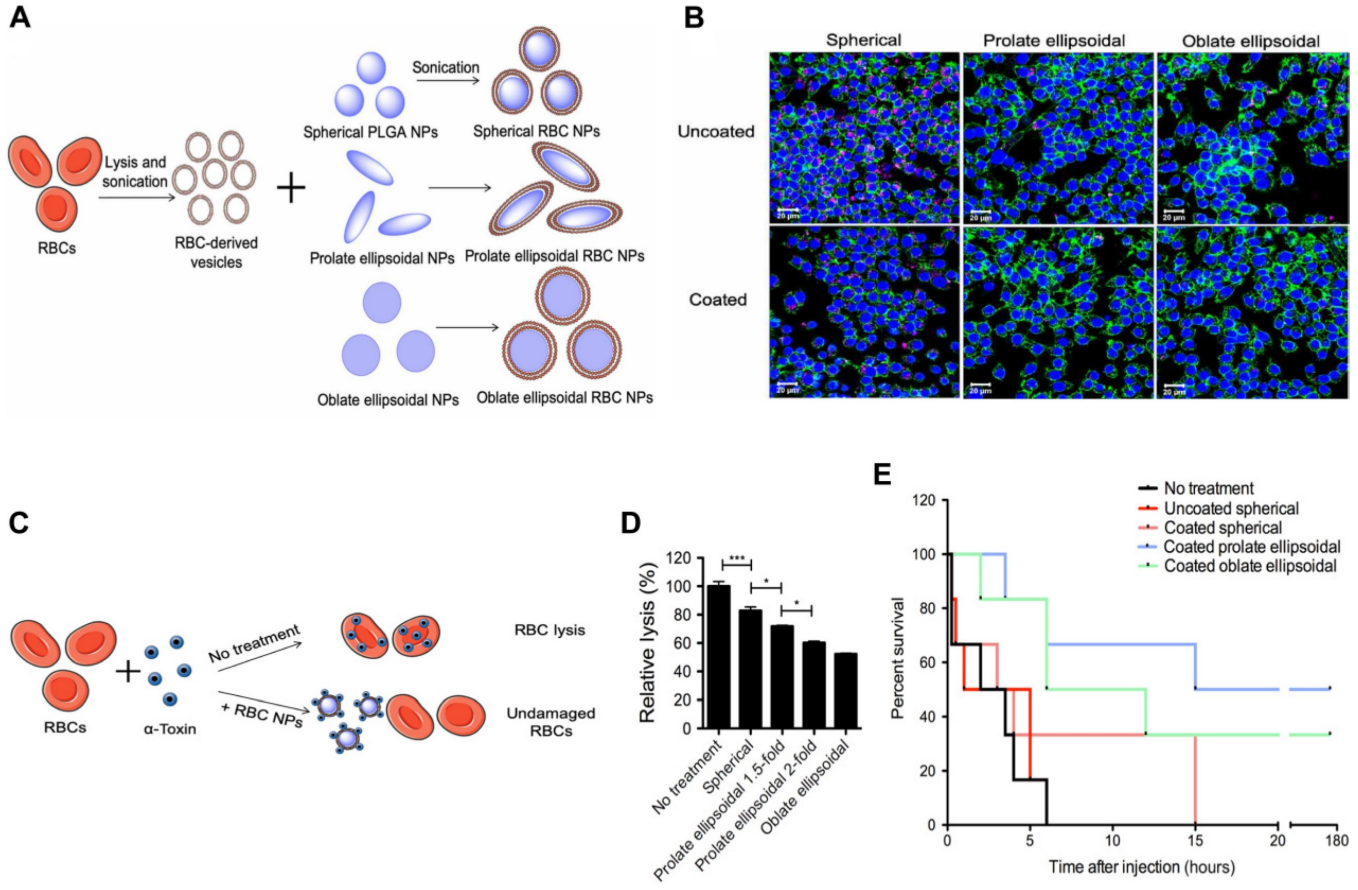
** Anisotropic RBC-NPs with the functions of enhanced circulation and toxin removal for sepsis therapy.** (**A**) Schematic representation of the production of anisotropic RBC-NPs. (**B**) Confocal imaging of pink-stained nanoformulations swallowed by macrophages with blue-stained nuclei and green-stained actin. Scale bar = 20 μm. (**C**) Schematic illustration of RBC-NP detoxification. (**D**) Quantification of RBC lysis to evaluate α-toxin absorption ability of listed RBC-NPs. (**E**) The survival rate of mice in the indicated treatment groups. ^*^P ≤ 0.05, ^***^P ≤ 0.001. Adapted with permission from 88, copyright Year 2020 American Association for the Advancement of Science Springer Nature.

To further enhance the circulation time and toxin removal potential of RBC-NP, Ben-Akiva *et al.* recently developed an innovative design 88. Non-spherical particles are able to avoid being removed from the body, resulting in an extended period of time in the system and improved therapeutic outcomes. Consequently, Ben-Akiva and his team fused RBC membranes onto anisotropic instead of spherical PLGA NPs to fabricate anisotropic RBC-NPs. Spherical nanoparticles were initially synthesized and then prolate or oblate ellipsoidal NPs were produced (**Figure 6A**). The taking up of both coated and uncoated nanoparticles by RAW 264.7 macrophages, an *in vitro* model of RES clearance, was assessed qualitatively using confocal microscopy (**Figure 6B**). Results demonstrated that the anisotropic shape of the NPs and the membrane coating combined could lead to improved immunity to macrophage destruction. Next, RBC lysis tests were conducted to assess the impact of the inner polymeric core shape alone, and the rate of hemolysis in the samples containing coated anisotropic particles was considerably lower than that of the coated spherical NPs (**Figure 6C-D**). After the promising results from the *in vitro* experiments, the detoxification capacity of the anisotropic NPs was assessed *in vivo*. Remarkably, mice treated with the oblate and prolate ellipsoidal anisotropic RBC-NPs showed a marked improvement in survival (**Figure 6E**). These results validate the anisotropic NPs as a powerful treatment option for sepsis patients compared to their spherical counterparts.

Beyond the absorption and neutralization of broad-spectrum toxins, RBC-NPs execute the function of 'on demand' antibiotic delivery. In one case, RBC membranes were used to hide supramolecular gelatin NPs (SGNPs) loaded with the antibiotic vancomycin (Van) to prepare an antibiotic administering system (Van⊂SGNPs@RBC) 89. *In vitro* experiments demonstrated that the shell of the RBC membrane serves as a protective barrier to reduce immune system clearance of antibiotics during delivery and absorb bacterial exotoxins to alleviate symptoms caused by infection. The core, containing cross-linked NPs, allows for the release of antibiotics in response to the bacterial infection environment. Although the multifunctionality of Van⊂SGNPs@RBC featuring environmentally sensitive antibiotic delivery and detoxification is not verified in the sepsis model, it is speculated that the system might be promising for treating bacterial sepsis.

### 2.3 Macrophage cell membrane-camouflaged nanoparticles (MΦ-NPs) as cellular nanosponges for the treatment of bacterial sepsis by concurrently neutralizing endotoxin and several types of inflammatory cytokines

Macrophages (MΦs) act as a primary line of defense against bacterial infection, containing a wealth of PRRs and cytokine-binding receptors. This suggests that they accumulate at sites of inflammation and effectively neutralize any inflammatory cytokines present 90. Therefore, MΦ-NPs have been widely studied for sepsis treatment.

**Figure 7:**
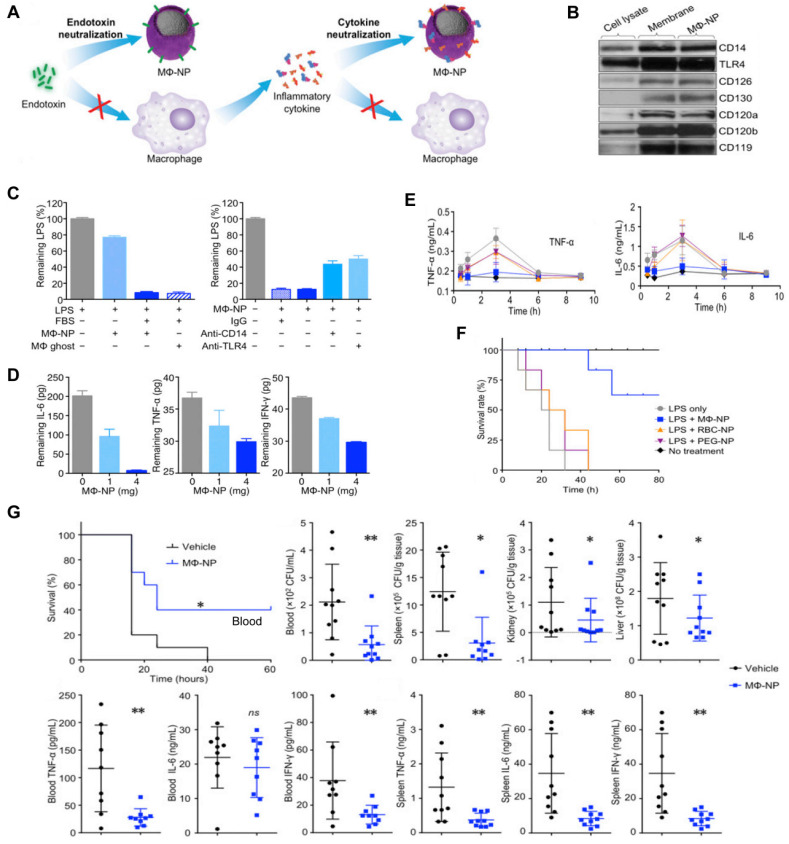
** MΦ-NPs concurrently absorb endotoxin and proinflammatory cytokines to treat sepsis.** (**A**) Schematic illustration of MΦ-NPs neutralizing endotoxin and proinflammatory cytokines. (**B**) Qualitative analysis of representative MΦ membrane proteins by Western blotting. (**C**) Left: the capacity of MΦ-NPs to remove LPS in the presence and absence of LPS binding protein. Right: non-specific IgG and antibodies that block CD14 and TLR4. (**D**) Removal of indicated proinflammatory cytokines with MΦ-NPs. (**E**) Levels of specified proinflammatory cytokines in plasma. (**F**) The survival rate of mice exposed to LPS who were given different forms of treatment. (**G**) Evaluation of the therapeutic efficacy of MΦ-NPs on mice by survival rate of mice, bacteria counts, and levels of proinflammatory cytokines. ^*^P ≤ 0.05, ^**^P ≤ 0.01. Adapted with permission from 91, copyright Year 2017 National Academy Sciences.

Thamphiwatana *et al.* first developed MΦ-NPs by coating PLGA NP with a cell membrane derived from MΦ 91. In these nanoplatforms, the MΦ membrane shell possesses an antigenic outer surface that is the same as the original macrophage cell; therefore, it can bind to the endotoxins. Concurrently, they act as decoys, binding to cytokines and blocking their ability to set off an inflammatory response (**Figure 7A**), for instance, the abnormal “cytokine storm”. These two features offer a type of treatment with significant potential for managing sepsis. Specifically, Western blot analysis revealed that MΦ-NPs still carry the vital membrane proteins which are responsible for LPS binding, such as CD14 and TLR4. Furthermore, cytokine receptors like CD126 and CD130 for IL-6, CD120a and CD120b for TNF, and CD119 for IFN-γ are also retained (**Figure 7B**). Furthermore, *in vitro* assays indicated MΦ-NPs could adhere to LPS (**Figure 7C**) and take proinflammatory cytokines such as IL-6, TNF, and IFN-γ (**Figure 7D**). *In vivo* experiments were conducted on mice injected with LPS to assess the inhibitory effect of MΦ-NPs on acute inflammatory responses to endotoxin and the LPS neutralization capacity of MΦ-NPs. Results showed that the levels of TNF-α and IL-6 in the blood did not increase in the MΦ-NP group, while the levels in other groups mirrored that of the LPS-only group (**Figure 7E**). Also, MΦ-NPs salvaged 60% of mice, while neither RBC-NP nor PEG-NPs made a significant difference in the survival rate of mice exposed to LPS (**Figure 7F**). Furthermore, the possible benefits of MΦ-NPs were studied using a live model of gram-negative bacterial sepsis. The results showed that only one dosage of MΦ-NPs had a significant survival benefit. The bacterial levels in the blood, spleen, kidney and liver of the mice treated with MΦ-NPs were significantly lower than those in the control group. Consequently, there was a notable diminishment of proinflammatory cytokines, such as IL-6, TNF-α, and IFN-γ, in both the bloodstream and spleen (**Figure 7G**).

**Figure 8:**
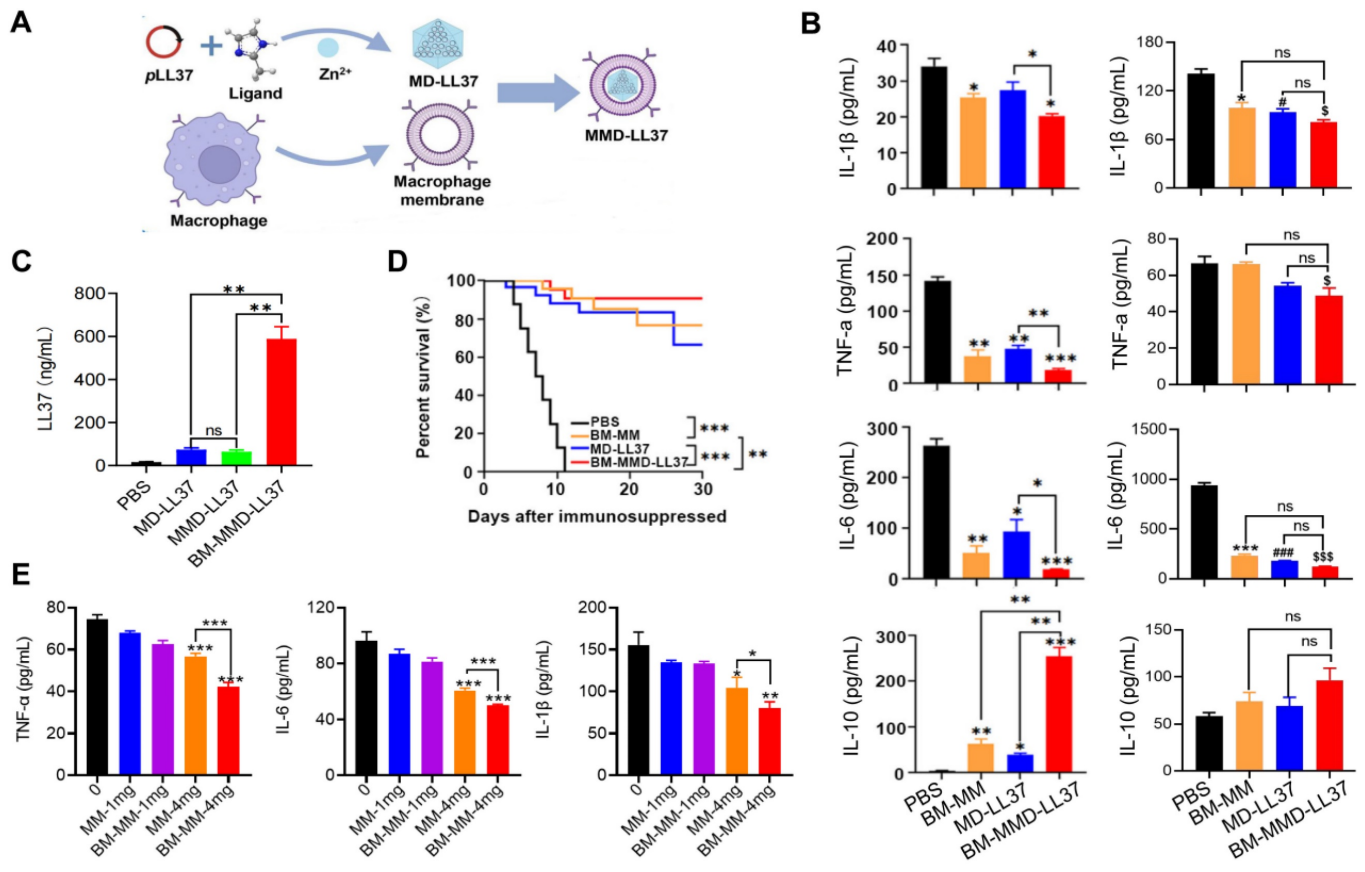
** MΦ membrane (MM)-concealed metal-organic framework (MOF) system simultaneously kills bacteria and absorbs proinflammatory cytokines to combat sepsis.** (**A**) Schematic illustration of the productionof MΦ membrane-concealed MOFs for the administration of pLL37 (MMD-LL37). (**B**) The levels of IL-6, TNF-α, IL-1, and IL-10 in the serum of immunocompromised mice with combined sepsis resulting from MDRSA and *E. coli* were examined 72 h after being administered various formulations. ^*^P < 0.05 versus MM, ^#^*P* < 0.05 versus MD-LL37, ^$^*P* < 0.05 versus MMD-LL37, ***P < 0.001 versus MM, ^###^*P* < 0.001 versus MD-LL37, ^$$$^*P* < 0.001 versus MMD-LL37. (**C**) The concentrations of IL-6, TNF-α, IL-1, and IL-10 in the serum of immunocompromised mice with sepsis caused by MDRSA were measured at 72 h after treatment with different formulations. (**D**) The level of LL37 in healthy mice after being exposed to various formulations for 48 h. (**E**) Survival rate of mice with sepsis after exposure to different formulations. (**F**) The effectiveness of 0 to 4 mg/mL MM or BM-MM to bind with and IL-1β, TNF-α, and IL-6. ^*^P < 0.05, ^**^P < 0.01, ^***^P < 0.001. Adapted with permission from 92, copyright Year 2022 American Chemical Society.

Cao *et al*. developed a MΦ Membrane (MM)-Camouflaged Metal-Organic Framework (MOF) system to tackle sepsis by delivering plasmid DNA (*p*DNA) 92. The MM coating allowed for the targeted delivery of the antimicrobial gene LL37 to MΦs, and the construction of MΦ factories to continuously generate antimicrobial peptides. Specifically, LL37 was placed in a MOF that was responsive to pH to produce MD-LL37, which was then masked with RAW 264.7-derived MM and MM from bone marrow (BM-MM) to form MMD-LL37 and BM-MMD-LL37, respectively. The effectiveness of MMD and BM-MMD for delivering the antimicrobial gene LL37 was assessed in healthy mice by measuring LL37 expression levels. The findings indicate that the cell membranes from primary MΦs have greater potential for masking the MOFs to facilitate efficient gene transfection *in vivo* than later generations of RAW 264.7 cells (**Figure 8A**). Furthermore, the ability of MMD-LL37 and BM-MMD-LL37 to take pro-inflammatory cytokines was investigated in immunodeficient septic mice. Evident modifications in cytokines that induce inflammation (IL-6, TNF-α, and IL-1β) and cytokines that inhibit inflammation (IL-10) were noticed in the BM-MMD-LL37 cluster (**Figure 8B**), but not in the MMD-LL37 cluster (**Figure 8C**), which were associated to proteins that are important to the functioning of cell membranes, such as TLR4, TNFR2, CD36, and CCR2 being on BM-MM and capturing and reducing pro-inflammatory cytokines. Furthermore, the effectiveness of MMD and BM-MMD for delivering the antimicrobial gene LL37 was tested in healthy mice by measuring the expression of LL37. The results suggested that the cell membranes of primary MΦs had greater success in concealing metal-organic frameworks (MOFs) to accomplish high levels of gene therapy delivery *in vivo* than successive passages of RAW 264.7 cells (**Figure 8D**). Also, BM-MMD-LL37 remarkably improved the survival rates of mice (**Figure 8E**). Finally, *in vitro* assays were performed to investigate the capacity of BM-MM and MM to take up pro-inflammatory cytokines. BM-MM had a better ability to bind to inflammatory factors than MM alone (**Figure 8F**). Overall, this research shows that BM-MMD-LL37 is able to effectively pass on the membrane proteins from the parent cells, enabling better sequestering of inflammatory cytokines and homologous targeting, which may be an effective way to treat sepsis.

Based on a similar working mechanism, other researchers constructed MΦ-NPs for treating LPS-induced endotoxemia. New MΦ-NPs had a notable effect on suppressing the immune reaction, lessening the inflammatory response, and increasing the survival rate of endotoxic mice, which demonstrated the potential of MΦ-NPs in the treatment of sepsis 93, 94. A brief overview of the MNPs mentioned above in sepsis treatment is summarised in **Table 1**.

## 3. Other promising MNPs for sepsis therapy

### 3.1 Hybrid MNPs

The hybrid formulation combines two different elements into one strong hybrid system that has its own distinct physical and biochemical characteristics 95. To enhance the capabilities and enable multitasking in intricate biological systems, hybrid MNPs with enhanced functionalization have been developed as follows.

The combination of cell membranes is the initial formulation. MNPs can be given properties similar to those of a source cell membrane. NP coated with cell membrane derived from single-cell type has limited functions; however, by combining two or more cell membranes, a hybrid approach can be utilized to add more features to the system. This fusion of membranes allows for more functions to be enabled. The resulting nanoformulation inherits the functions of all source cell membranes 96.

**Figure 9:**
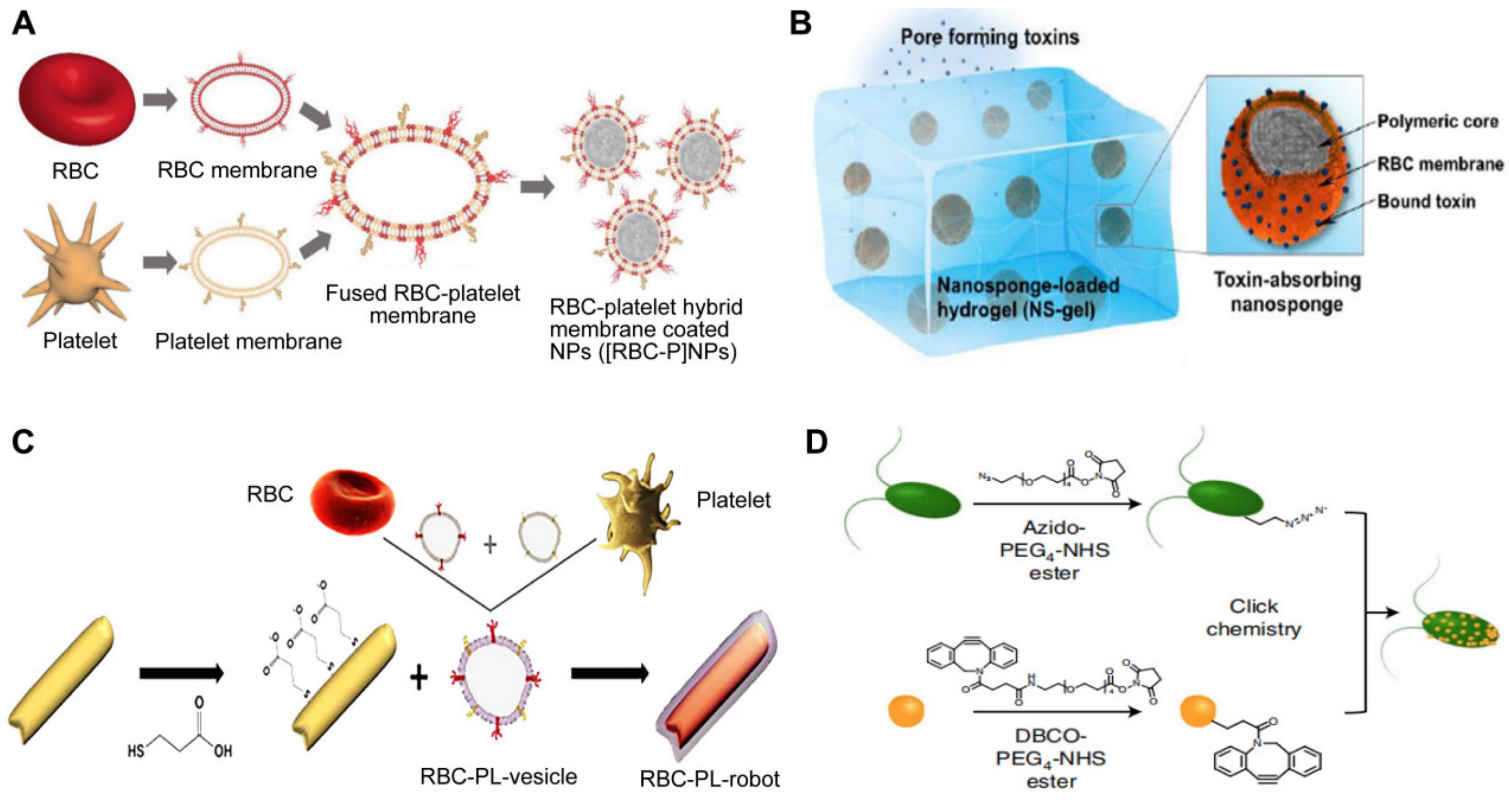
** Four types of hybrid MNPs.** A diagram is presented that demonstrates the process of preparation of [RBC-P]NPs (**A**), RBC-PL-robots (**B**), hydrogel-retaining toxin-absorbing nanosponges (**C**) and algae-NP-robots (**D**). Adapted with permission from 97, 98, 100, and 101, copyright Year 2017 Wiley-Blackwell, Year 2018 American Association for the Advancement of Science, Year 2015 Wiley-Blackwell, and Year 2022 Springer Nature, respectively.

RBC-PL hybrid membrane-coated nanoparticles ([RBC-P]NPs) (**Figure 9A**), may stay in the blood for a longer time and target specific tissue due to the introduction of PL membrane with a targeting ligand 97. A study developed nanorobots without fuel that have been functionalized with a hybrid membrane composed of RBCs and PLs. These nanorobots, referred to as “RBC-PL-robots”, were made by encasing acoustic AuNW robots with the hybrid membrane (**Figure 9B**) 98. *In vitro* experiments demonstrated these biomimetic nanorobots' dual detoxification ability to attach to and contain pathogens that adhere to surfaces and neutralize bacterial toxins. Apart from the hybrid of the RBC membrane and PL membrane, MΦ-PL, PL-leukocyte, and PL-cancer stem cells were combined for a variety of applications, such as combination treatments and individualized cancer therapy 99.

The alternatively hybrid formulation is the combination of distinct materials. For example, Wang *et al.* integrated toxin nanosponges with hydrogel for the localized management of bacterial contamination (**Figure 9C**) 100. The nanosponges are able to absorb and divert PFTs away from their intended targets, while the hydrogel serves to retain them at the infection sites, facilitating localized toxin neutralization for enhanced therapeutic potency. The combined advantages of the nanosponge-hydrogel formulation were confirmed by the significantly decreased MRSA skin lesion in a mouse model. More recently, Zhang *et al*. created microscopic robots designed to administer antibiotics in an active manner directly into the lungs *in vivo* by using click chemistry to adhere NPs coated with a neutrophil membrane loaded with antibiotics to natural microalgae (designated as algae-NP-robot) (**Figure 9D**) 101. The hybrid microrobot combined the motility of *Chlamydomonas reinhardtii* microalgae and cell-mimicking properties of neutrophil membrane-coated NPs, for instance, it featured protective barriers for payloads from biological settings, decreased the possibility of being disposed of by the immune system and allowed for attachment to a particular target pathogen. In a mouse research investigation into the effects of acute Pseudomonas aeruginosa pneumonia, algae-NP robots were successful in reducing bacterial load and significantly improving survival rates. This demonstrated the potential of these microrobots for actively delivering treatments to the lungs, especially in intensive care settings.

Despite that the fused membrane unites the benefits of its parent membranes and has shown to be exceptionally effective in therapeutic applications compared to the same monotypic cell membrane type, there is no report of sepsis treatment. Therefore, some thoughts and improvements are proposed to exploit hybrid MNPs for the treatment of sepsis in the future. First, given that many pathogenic factors are involved in sepsis, the hybrid of three or more cell membranes will work better than when two cell membranes are used. Second, with various cell membranes chosen to fuse, new large-scale culture techniques are needed to acquire large quantities of parent cells. In addition, the relative quantities of the different kinds of membrane to fuse must be considered carefully based on the application of specific sepsis. Last, there is an urgent need to improve the precision of hybrid MNPs, which may be endowed by new proteins introduced to natural source cell membranes through artificial ways, as shown in the following section of engineered MNPs.

### 3.2 Engineered MNPs

Modifying the natural cell membranes directly or indirectly is a technique known as membrane engineering. Despite the natural characteristics of cell membranes, they have yet to reach some desired functionalities 102. Consequently, NP wrapped with engineered cell membranes may add new functions that natural cell membranes cannot supply.

**Figure 10:**
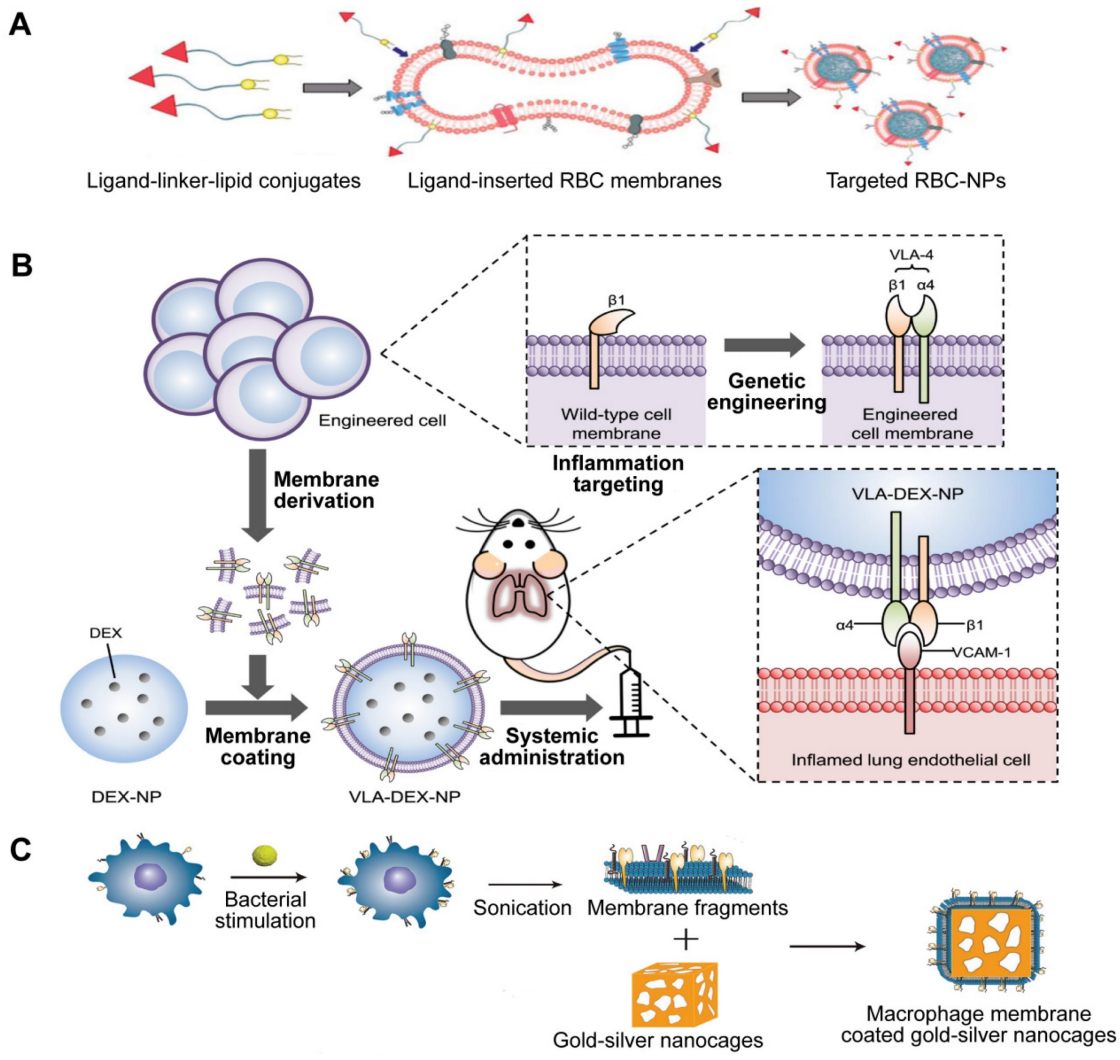
**Three types of engineered MNPs.** Schematic illustration of the preparation of targeted RBC-NPs (**A**), genetically engineered MNPs (**B**), and Sa-M-GSNCs (**C**). Adapted with permission from 104, 105, and 106, copyright Year 2013 Royal Society of Chemistry, Year 2021 American Association for the Advancement of Science, and Year 2018 Wiley-Blackwell, respectively.

The alteration of cellular membranes focuses on attaching specific ligands to the external coating of the cell membrane in order to target certain receptors of the intended cell through physical or chemical means 103. Utilizing lipids as a means of physical modification is a widespread approach due to its easy implementation and adjustable impact on the object being modified. For instance, NPs encapsulated with RBC membranes have a longer half-life in circulation but lack target selectivity that promises the reduction of unintended effects. Fang et al. used the lipid-insertion method to add the desirable feature to produce functionalized RBC membranes (**Figure 10A**) 104. Following the introduction of two different sizes of ligands, a small molecule folic acid and an aptamer called AS1411 that targets nucleolin, the RBC-NPs demonstrated a specific affinity for cancer cell lines.

Indirect alterations can be accomplished by manipulating the existing biosynthetic pathways or modifying the genes of the cell to make the cell membrane functionalized through metabolic and genetic engineering techniques. Park* et al.* genetically engineered MNPs for directed delivery of dexamethasone (DEX) to lungs with inflammation (**Figure 10B**) 105. Inflamed endothelial cells can increase the amount of vascular cell adhesion molecule-1 (VCAM-1) they express in order to draw in leukocytes that possess the complementary very late antigen-4 (VLA-4). By introducing VLA-4 into C1498 mouse leukemia cells, the researchers created C1498-VLA cells. The engineered membrane from these cells was then utilized to cover DEX-loaded polymeric NPs, a potent anti-inflammatory drug. The therapeutic efficacy of the final nanoformulations (denoted as VLA-DEX-NPs) was assessed *in vivo* via a mouse model of lung inflammation brought on by endotoxin, and the results showed better administration of the drug load to lungs with inflammation and considerable therapeutic effectiveness.

Similarly, based on the potential interaction of receptors and ligands, Wang *et al.* designed a new targeting delivery nanosystem by camouflaging gold-silver nanocages (GSNC) with pretreated MΦ membranes (**Figure 10C**) 106. It is known that PRRs on the MΦ membrane are responsible for recognizing microbial pathogens by binding to their ligands PAMPs, and the expression of PRRs will increase when MΦ is activated by bacteria 107. Thus, they first exposed MΦ to *S. aureus* and *E. coli* and established that the levels of PAMPs on the MΦ membranes were up-regulated. Then, they prepared M-GSNCs, Ec-M-GSNCs, and Sa-M-GSNCs by coating GSNCs with the MΦ membranes, *E. coli*-pretreated MΦ membrane, and *S. aureus*-pretreated MΦ membrane, respectively. The Sa-M-GSNC nanosystem showed improved delivery and retention at the site of *S. aureus* infection when administered via local or systemic injections compared to PBS, GSNCs, M-GSNCs, and Ec-M-GSNCs, indicating that the novel nanosystem is able to deliver its payload specifically to the target bacteria.

Membrane engineering eliminates the boundaries of what is achievable with a natural membrane, opening up a world of potential for applying engineered MNPs to sepsis. However, there is much room for improvement. First, because gene alteration is a complex procedure, it is difficult for the engineered membrane to guarantee the consistent expression of certain target genes, it is important to keep their expression levels stable. Therefore, incorporating new strategies such as electro-transformation or transposon-mediated transfection can significantly enhance the safety and efficiency of genetic engineering. Second, modifying living cells' membranes prior to removal is superior to directly changing isolated cell membranes because the latter can lead to a malfunction of the membrane. The customisation of engineered MNPs is not only limited to membrane coating, but also involves altering the nucleus and membrane, which can have a synergistic effect on their multi-functionality. Lastly, the combination of membrane engineering and membrane fusion is perceived to achieve the desired multifunctionality of cell membranes.

### 3.3 Antivirulence vaccines based on MNP

Vaccines that are approved for use have proven to be an effective way to limit the need for antibiotics, ultimately reducing healthcare expenses and decreasing the number of bacterial strains that are resistant to drugs. Vaccination, based on toxin neutralization rather than the cytotoxic activity of antibiotics, can inhibit pathogens from colonizing hosts, which does not directly exert pressure on the individual bacterium and thus leads to the genetic variation of drug resistance 108. Although attractive and safe, vaccines that are able to protect against a wide variety of infectious bacterial diseases are currently unavailable. This includes *Streptococcus pyogenes*, *S. aureus*, *Helicobacter pylori*, *Chlamydia, Shigella*, *E. coli*, and many other bacteria-caused infections. This is due to the following challenges in designing antivirulence vaccines: first, creating vaccines against biological toxins requires knowing toxin's function in advance and removing the toxin either from a natural origin or a recombinant one. Occasionally, even full knowledge of a toxin does not guarantee its application to vaccine design. Second, patients with sepsis are challenged by different bacterial species and strains, which secrete various toxins, while most vaccines only train the immune system against one antigen 109. Finally, the balance between safety and immunogenicity must be disturbed, which often exhibits an inverse relationship 110.

**Figure 11:**
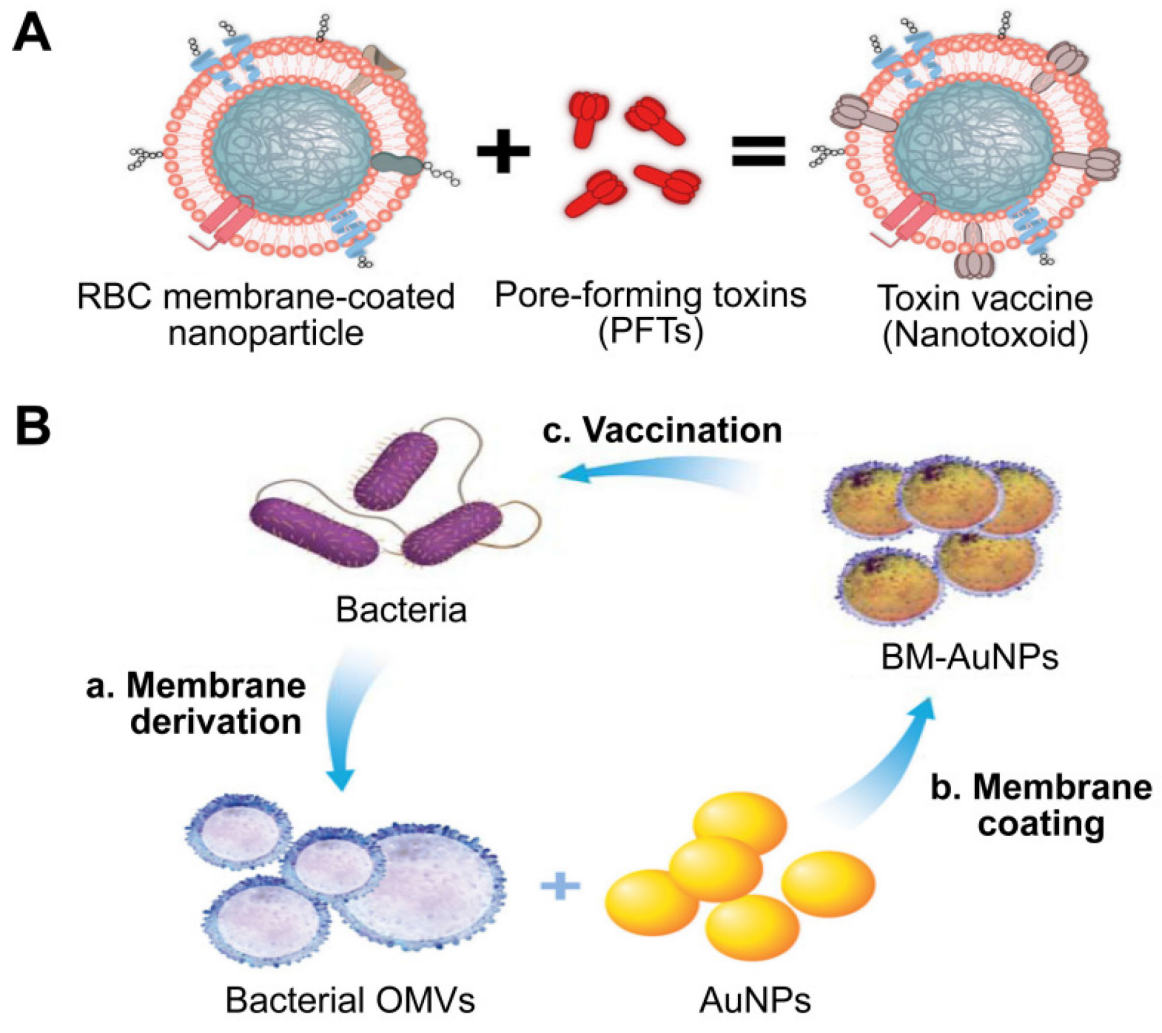
**Two types of antivirulence vaccines based on MNPs.** Schematic illustration of the (**A**) the preparation of nanotoxoid and BM-AuNPs vaccine (**B**). Adapted with permission from 111 and 113, copyright Year 2013 Springer Nature, and Year 2015 American Chemical Society, respectively. AuNPs: gold nanoparticles; BM-AuNPs: bacterial membrane-coated AuNPs; OMVs: outer membrane vesicles.

Given that cell membrane has a natural affinity for multiple bacterial toxins and efficiently neutralizes the membrane-damaging activity of PFTs, MNPs bound to harmful toxins can safely deliver the toxins back to the immune system in the form of a “nanotoxoid” to generate antibacterial immunity (**Figure 11A**) 111. This novel vaccine strategy addresses the above hurdles in the design of antivirulence vaccines. For example, the α-hemolysin (Hla)-loaded nanotoxoids, known as nanotoxoid (Hla), were prepared by mixing RBC-NP with staphylococcal Hla 112. Immunization studies verified that the nanotoxoid (Hla) could elicit Hla-specific antibodies. Furthermore, antibody quantification results showed that the nanotoxoid (Hla) was able to elevate Hla-specific antibody titers in comparison to heat-treated Hla. Furthermore, *in vivo* experiments demonstrated that the Hla vaccine can enhance immunity in vaccinated mice, which received a lethal dose of Hla through the tail mainline. Wei *et al.* prepared nanotoxoid by incubating RBC-NP with a hemolytic secreted protein (hSP) fraction collected from culture supernatant of MRSA strain USA300 110. Titer analysis of the α-toxin, Panton-Valentine leukocidin, and γ-toxin showed that the nanotoxoid (hSP) formulation was superior in enhancing anti-toxin immune responses to the heat-treated hSP formulation. After challenge for 3 days, mice vaccinated with nanotoxoid (hSP) could effectively remove MRSA bacteria compared to those receiving heat-treated hSP.

Gao *et al.* introduced a unique bacterial membrane-coated nanoparticle system as a new and exciting antibacterial vaccine (**Figure 11B**) 113. They fabricated bacterial membrane-coated AuNPs (BM-AuNPs) by coating bacterial outer membrane vesicles (OMVs) onto small gold nanoparticles (AuNPs) with a diameter of 30 nm. The bacterial membranes and the AuNP cores mutually benefited each other, synergistically generating enhanced antibacterial immune responses.

## Conclusions and Prospects

The biggest advantage of MNPs in the treatment of sepsis is their multifunctionality, conferred by integrating cell membranes and NPs. They can deliver antibacterial and anti-inflammatory drugs; they offer good biocompatibility, i.e., the “self” property of cell membranes makes MNPs exert desired functions without any undesired local or systemic effects during a particular application; they have targeting capability, which improves the biodistribution and bioavailability of MNPs; they can absorb and neutralize broad-spectrum toxins and/or inflammatory factors as decoys. The combination significantly increases the therapeutic effect when fighting single factors or/and multiple factors associated with sepsis. In addition, other approaches based on MNPs, such as hybrid MNPs, engineered MNPs, and nanotoxoids, show great promise for sepsis therapy in the future.

Despite thriving development, further optimization of MNPs is urgently needed to treat sepsis. First, multifunctionalization of MNPs for treating bacteria sepsis is still in an early stage. Second, the design and utilization of almost all MNPs still rely on laboratory experimentation due to various technical limitations, such as immature isolation, purification methods, low yields, and insufficient loading and delivery efficiency with therapeutic payloads. Third, thorough characterization of NPs is critical to increasing the quality, efficacy, and safety of NPs, which is a prerequisite for a broad common acceptance of novel nanotechnology in the public 114. NPs consist of a variety of materials, such as polymeric (PLGA, PEG), lipid-based (liposomes, lipid NPs), inorganic (gold, silica), or biologically derived (cell-membrane vesicles) inorganic materials, which have unique properties compared with the bulk phase and allow for a wide variety of possible structures and characteristics 115. The characterization of NPs includes size, shape, composition, surface charge, drug release kinetics, stability, and toxicity 116. However, the lack of accurate and reliable characterization techniques and their standardization is the main bottleneck of NP characterization 117.

Despite extensive challenges, MNPs hold great promise and have great prospects for treating sepsis. First, in addition to intervening in pathogenic bacteria, toxins, and inflammatory cytokines, MNPs offer new opportunities for immune modulation, which is the core mechanism underlying sepsis occurrence and development. The number of patients with sepsis who die of early inflammation is declining due to improved surveillance and advances in supportive care, while mortality among patients in the late stages of sepsis who are immunosuppressed is increasing. The reversal of immunosuppression is a topic of intense research among numerous laboratories worldwide 118-120. However, most nanomaterials currently focus on the first stage of sepsis (excessive activation of the inflammatory response) and rarely involve the late stage. Additionally, antibiotics are often ineffective and have little impact on lowering the mortality rate in immunosuppression. Therefore, developing MNPs that can reverse sepsis-induced immunosuppression by loading immune agonists into NPs will be an attractive immunotherapy. Furthermore, sepsis is accompanied by the balance deviation of M1/M2 MΦ. In the early stage of sepsis, M1-like MΦs continue to increase in number and cause severe inflammatory responses. Conversely, an excessive increase in M2-like MΦs during late-stage sepsis induces an immunosuppressive state in the host 121. A recent study reported that hybrid cell membrane nanovesicles promote repolarization of M2-to-M1 within the immunosuppressive tumour microenvironment 122. Thus, targeted regulation of macrophage polarization by MNPs will open up new therapeutic avenues for sepsis and related diseases. Second, in addition to therapy, rapid, sensitive and specific detection of infectious pathogens is also crucial for the clinical progression and outcome of a septic patient. Some NPs have been developed for the diagnosis and theranostics of sepsis, and can be further optimized with cell membrane coatings 123. Third, in the process of MNP fabrication, such approaches as antibiotic payload, membrane fusion, and membrane modification are compatible. Any combination is available whenever necessary, achieving the desired multifunctionalization and thus can target multiple pathogenic factors in sepsis. Finally, as the research moves forward, innovative methods of MNP fabrication will be added. For example, it becomes increasingly interesting and important to develop biomimetic nanorobots that are prepared by coating self-propelled and autonomous nanomotors; the propulsion enhances the binding and detoxification efficiency of the robots against pathogens and toxins. The introduction of intelligence capabilities to MNPs will be a breakthrough for sepsis therapy.

Overall, MNPs are currently viewed as an attractive therapeutic strategy that overcomes challenges associated with sepsis management due to their inherent multifunctionality-able to invade the clearance by the immune system, conquer bacterial resistance, and neutralize a broad spectrum of toxins and inflammatory factors. Studies of MNP biology and a comprehensive understanding of the challenges and limitations of sepsis therapy are expected to lay the solid foundation for future clinical application.

## Supplementary Material

Supplementary tables.

## Figures and Tables

**Table 1:** Types of MNPs for sepsis treatment.

MNP types	Membrane derivation	Core particles	Sepsis models	MNP functions	Refs (Publication date)
PNP-Van	PL	Spherical loaded-Van PLGA	A mouse model of systemic MRSA252 infection	Targeted antibiotic delivery for the binding of PL to pathogen; biocompatibility	76 (2015)
RBC membrane- coated PLGA NP	RBC	Spherical PLGA	A lethal dose of α-toxin induced acute death in mice	Prolonged blood circulation; biocompatibility; deflecting the intensity of PFTs is a way to avoid their harmful effects.	82 (2013)
RBC-NS	RBC	Spherical PLGA	A sublethal dose of MRSA wSP induced lethality in mice	Neutralizing the hemolytic activity of MRSA wSP	86 (2019)
Anisotropic RBC membrane-coated NP	RBC	Anisotropic PLGA	A lethal dose of alpha toxin induced mouse sepsis model	Increasing half-life; enhancing activity of alpha toxin absorption	88 (2020)
MΦ-NP	MΦ	Spherical PLGA	Bacterial sepsis model induced by *E. coli*	Neutralizing endotoxins; sequestering proinflammatory cytokines; reducing bacterial burden	91 (2017)
MΦ-NP	MΦ	Loaded-*p*DNA (LL37) MOF	Immunosuppressed mice with mixed sepsis induced by MDRSA and *E. coli*	Targeted *p*DNA (LL37) delivery; sequestering inflammatory cytokines	92 (2022)
Fe_3_O_4_@MM	MΦ	Fe_3_O_4_	LPS-induced mouse sepsis model	Deactivating LPS; biocompatibility; sequestering proinflammatory cytokines	93 (2019)
PEG-Mac@NP	MΦ	PEG	LPS-induced mouse endotoxemia model	Neutralizing proinflammatory cytokines and endotoxins	94 (2020)

*E. coli*: *Escherichia coli*; LPS: lipopolysaccharide; MM: MΦ membrane; MNP: cell membrane-coated nanoparticles; MOF: metal-organic framework; MRSA252: *Methicillin resistant Staphylococcus aureus 252*; NP: nanoparticle; *p*DNA: plasmid DNA; PEG: polyethylene glycol; PFT: pore-forming toxin; PL: platelet; PLGA: poly (lactic-co-glycolic acid); PNP: Platelet membrane-cloaked NP; RBC: red blood cell; RBC-NS: RBC nanosponge; Van: vancomycin; wSP: whole secreted protein.

## Abbreviations

ALRs: AIM-2 like receptors
AuNPs: gold nanoparticles
BM-AuNPs: bacterial membrane-coated AuNPs
MNPs: cell membrane-coated nanoparticles
CD: cluster of differentiation
DEX: dexamethasone
DAMPs: danger-associated molecular pattern molecules
DIC: disseminated intravascular coagulation
GSNCs: gold-silver nanocages
HSP: heat-shock protein
hSP: hemolytic secreted protein
HMGB1: high-mobility group box 1
IFN: interferon
IL: interleukin
LPS: lipopolysaccharide
MΦ: macrophage
MOF: metal-organic framework
NP: nanoparticle
NLRs: NOD-like receptors
OMVs: outer membrane vesicles
PAMPs: pathogen-associated molecular pattern molecules
PEG: polyethylene glycol
PFTs: pore-forming toxins
PL: platelet
PLGA: poly (lactic-co-glycolic acid)
PNPs: platelet membrane-cloaked nanoparticles
PRRs: pattern recognition receptors
RBC: red blood cell
RES: reticuloendothelial system
RONS: reactive oxygen and nitrogen species
SIRPα: signal regulatory protein alpha
SGNPs: supramolecular gelatin nanoparticles
TLRs: toll-like receptors
TNF: tumor necrosis factor
Van: vancomycin
VCAM-1: vascular cell adhesion molecule-1
VLA-4: very late antigen-4
