# Supplementary material for: Cell-membrane-coated nanoparticles for the fight against pathogenic bacteria, toxins, and inflammatory cytokines associated with sepsis
Source: Theranostics. 2023 May 21;13(10):3224–44. doi: 10.7150/thno.81520 (PMC10283065; doi:10.7150/thno.81520)
Supplement: Supplementary file 1 — Supplementary tables. [file thnov13p3224s1.pdf]

Table S1. Treatment strategies directed at pathogenic bacteria in sepsis.

**Treatment strategies**	**Mechanisms of action**	**Refs (Publication date)**
Free antibiotics	Interfering with metabolic processes of bacteria	[1-3] (2021, 2016, 2020)
Antibiotic loaded NPs	Increasing antibiotic potency due to improved pharmacokinetics	[4] (2021)
Metal and metal oxide NPs	The disruption of the bacterial cell membrane, denaturation of protein, and DNA damage and disruption of the respiratory chain	[4] (2021)
Free AMPs	Physically destroying the lipid bilayers of bacteria	[5, 6] (2021, 2020)
Self-assembling chimeric peptide NPs with PEGylation	Alleviating the pharmacokinetic defect of peptide-based antibacterial drugs with the improved cytocompatibility	[7] (2022)
Liposome with a combined AMP and antibiotic	Synergism with significant improvement of bacterial clearance	[8] (2015)
Lipidic nanocapsules encapsulating essential oils	Antimicrobial activity of phenolic, aldehyde and alkene compounds in essential oils	[9, 10] (2016, 2016）

AMP: antimicrobial peptide; PEG: polyethylene glycol; NP: nanoparticle.

Table S2. Treatment strategies directed at toxins in sepsis.

Treatment strategies	Mechanisms of action	Refs (Publication date)
AMPs	Binding to LPS and LP	[11] (2018)
Gas gangrene antitoxin	Neutralizing a-toxin secreted by *C. perfringens*	[12] (2018)
Anti-endotoxin monoclonal antibody	Binding to endotoxin	[13] (2000)
J5 antiserum	Binding to LPS core	[14] (2000)
J5 immune plasma	Binding to LPS core	[14] (2000)
Intravenous immunoglobulins	Nonspecifically neutralizing bacterial toxins	[15] (2013)
Polymyxin B fiber column	Removing circulating endotoxin by adsorption	[16] (2017)
HDL like NPs	Scavenging and neutralizing LPS	[17] (2016)
Engineered liposomes	Sequestering bacterial toxins as decoy	[18] (2015)

AMP: antimicrobial peptide; HDL: High-density lipoprotein; LP: lipoprotein; LPS: lipopolysaccharide.

Table S3. Treatment strategies directed at inflammatory cytokines in sepsis.

Treatment strategies	Mechanisms of action	Refs (Publication date)
CytoSorb	Nonspecifically eliminating many proinflammatory cytokines	[16] (2017)
TLR4 Inhibitor	Inhibiting TLR4 signaling	[16] (2017)
APS loaded NPs	Inhibiting the activity of TLR4/NF-κB pathway with improved pharmacokinetics	[19] (2000)
Anti-TNF monoclonal antibody	Antibody-specific inactivation of circulating TNF	[20-22] (2003, 2001, 2001)
Soluble TNF receptors	Receptor-specific inactivation of circulating TNF	[20-22] (2003, 2001, 2001)
IL-1 receptor antagonists	Receptor-specific inactivation of IL-1	[20-22] (2003, 2001, 2001)
PAF receptor antagonist	Receptor-specific inactivation of PAF	[20-22] (2003, 2001, 2001)
Bradykinin inhibitor	Inhibiting the kallikrein/kinin cascade	[20-22] (2003, 2001, 2001)
Ibuprofen	Inhibiting the production of prostaglandin	[21, 22] (2001, 2001)
Glucocorticoids	Inhibiting the synthesis of almost all proinflammatory cytokines	[23, 24] (2002, 2005)
Cerium oxide NPs	Decreased transcriptional action of ROS, iNOS, COX-2, and NF-κB	[25] (2018)
Curcumin-loaded solid lipid NPs	Suppressing NF-kB activation and IkBa degradation levels	[26] (2015)
Bone marrow stromal cells	Reprograming host macrophages to increase their IL-10 production	[27] (2009)

APS: astragalus polysaccharide; COX-2: cyclooxygenase-2; IkBa: NF-kappa-B inhibitor alpha; IL: interleukin; iNOS: inducible nitric oxide synthase; NF-κB: nuclear factor kappa-B; NP: nanoparticle; PAF: platelet-activating factor; ROS: reactive oxygen species; TLR4: toll-like receptor 4; TNF: tumor necrosis factor.

Table S4. Some of transmembrane PRRs and corresponding PAMPs and DAMPs participating in the inflammation in sepsis.

**PAMPs/DAMPs**	**TLRs**	**Roles in inflammation**
Triacyl lipopeptide soluble factors	TLR1	
Lipoproteins	TLR2	
Matrix metalloproteinase 2	TLR2	
Peptidoglycan	TLR2	
mRNA	TLR3	
Fibrinogen	TLR4	
Envelope proteins	TLR4	
Heparan sulfate	TLR4	
Atypical LPS, LPS	TLR2 / TLR4	Producing proinflammatory cytokines
Hsp60, hsp70,hsp72, hsp22	TLR2 / TLR4	
Glycoprotein 96	TLR2 / TLR4	
Hyaluronic acid	TLR2 / TLR4	
HMGB1	TLR2 / TLR4 / TLR5	
Di-acyllipopeptides from mycoplasm	TLR2 / TLR6	
Amyloid β	TLR2 / TLR6	
Oxidized LDL	TLR2 / TLR6	
CpG-containing DNA from bacteria, host DNA from dying cells, mitochondrial DNA (mtDNA),	__	
HMGB1-DNA complex	__	
Flagellin	TLR5 / TLR11	

Note: "__" represents no.

HMGB1: high-mobility group box 1; hsp: heat-shock protein; LDL: low-density lipoprotein; LPS: lipopolysaccharide; TLR: Toll-like receptor.

Table S5. Some of cytoplasmic PRRs as well as corresponding PAMPs and DAMPs engaged in the inflammation in sepsis.

PAMPs	DAMPs	Intracellular PRRs	Roles in inflammation
g-D-glutamyl-mesodiaminopimelic acid (bacterial peptidoglycans)	__	NOD1	
muramyl dipeptide (bacterial peptidoglycans)	__	NOD2	
Bacillus anthracis lethal toxin		NLRP1	
Staphylococcus aureus, Candida albicans, Saccharomyces cerevisiae, Listeria monocytogenes, Neisseria gonorrhoeae, pore-forming toxins	Uricacid crystals, extracelluar ATP, HMGB1, hsp70, hsp90	NLRP3	Activating casp-1 causes pyroptosis
Listeria monocytogenes, Salmonella typhimurium, Shigella flexneri, Legionella pneumophila, Pseudomonas aeruginosa, Cytosolic flagellin	__	NLRC4	
Double-stranded DNA from Francisella tularensis	__	AIM2	
Cytosolic LPS	__	Cytosolic LPS sensor	Activating casp-11 causes pyroptosis

Note: “__” represents no.

ATP: adenosine triphosphate; hsp: heat-shock protein; LPS: lipopolysaccharide.

Supplemental References

1. Ulldemolins M, Bastida C, Llauradó-Serra M, Chantal Csajka C, Rodríguez A, Badia JR, et al. Once-daily 1 g ceftriaxone optimizes exposure in patients with septic shock and hypoalbuminemia receiving continuous veno-venous hemodiafiltration. Eur J Clin Pharmacol. 2021; 77: 1169-80.

2. Tsai D, Stewart P, Goud R, Gourley S, Hewagama S, Krishnaswamy S, et al. Optimising meropenem dosing in critically ill Australian Indigenous patients with severe sepsis. Int J Antimicrob Agents. 2016; 48: 542-6.

3. Masich AM, Kalaria SN, Gonzales JP, Heil EL, Tata AL, Claeys KC, et al. Vancomycin pharmacokinetics in obese patients with sepsis or septic shock. Pharmacotherapy. 2020; 40: 211-20.

4. Pant A, Mackraj I, Govender T. Advances in sepsis diagnosis and management: a paradigm shift towards nanotechnology. J Biomed Sci. 2021; 28: 6.

5. Chai JW, Chen X, Ye TF, Zeng BS, Zeng QY, Wu JN, et al. Characterization and functional analysis of cathelicidin-MH, a novel frog-derived peptide with anti-septicemic properties. ELife. 2021; 10: e64411.

6. Nagaoka I, Tamura H, Reich J. Therapeutic potential of cathelicidin peptide LL-37, an antimicrobial agent, in a murine sepsis model. Int J Mol Sci. 2020; 21: 5973.

7. Tan P, Tang Q, Xu SR, Zhang YC, Fu HY, Ma X. Designing self-assembling chimeric peptide nanoparticles with high stability for combating piglet bacterial infections. Adv Sci. 2022; 9: e2105955.

8. Fan X, Fan J, Wang X, Wu P, Wu G. S-thanatin functionalized liposome potentially targeting on Klebsiella pneumoniae and its application in sepsis mouse model. Front Pharmacol. 2015; 6: 249.

9. Swamy MK, Akhtar MS, Sinniah UR. Antimicrobial properties of plant essential oils against human pathogens and their mode of action: an updated review. Evid Based Complement Alternat Med. 2016; 2016: 3012462.

10. Montagu A, Joly-Guillou ML, Guillet C, Bejaud J, Rossines E, Saulnier P. Demonstration of the interactions between aromatic compound-loaded lipid nanocapsules and Acinetobacter baumannii bacterial membrane. Int J Pharm. 2016; 506: 280-8.

11. Heinbockel L, Weindl G, Martinez-de-Tejada G, Correa W, Sanchez-Gomez S, Bárcena-Varela S, et al. Inhibition of lipopolysaccharide- and lipoprotein-induced inflammation by antitoxin peptide Pep19-2.5. Front Immunol. 2018; 9: 1704.

12. Hifumi T, Nakano D, Chiba J, Takahashi M, Yamamoto A, Fujisawa Y, et al. Combined therapy with gas gangrene antitoxin and recombinant human soluble thrombomodulin for Clostridium perfringens sepsis in a rat model. Toxicon. 2018; 141: 112-7.

13. Angus DC, Birmingham MC, Balk RA, Scannon PJ, Collins D, Kruse JA, et al. E5 murine monoclonal antiendotoxin antibody in gram-negative sepsis: a randomized controlled trial. E5 Study Investigators. JAMA. 2000; 283: 1723-30.

14. Freeman BD, Natanson C. Anti-inflammatory therapies in sepsis and septic shock. Exp Opin Invest Drugs

2000; 9: 1651-63.

15. Alejandria MM, Lansang MAD, Dans LF, Mantaring III JB. Intravenous immunoglobulin for treating sepsis, severe sepsis and septic shock. Cochrane Database Syst Rev. 2013; 2013: CD001090.

16. Rello J, Valenzuela-Sánchez F, Ruiz-Rodriguez M, Moyano S. Sepsis: A review of advances in management. Adv Ther. 2017; 34: 2393-411.

17. Foit L, Thaxton CS. Synthetic high-density lipoprotein-like nanoparticles potently inhibit cell signaling and production of infammatory mediators induced by lipopolysaccharide binding Toll-like receptor 4. Biomaterials. 2016; 100: 67-75.

18. Henry BD, Neill DR, Becker KA, Gore S, Bricio-Moreno L, Ziobro R, et al. Engineered liposomes sequester bacterial exotoxins and protect from severe invasive infections in mice. Nat Biotechnol. 2015; 33: 81-8.

19. Xu X, Rui S, Chen C, Zhang G, Li Z, Wang J, et al. Protective effects of astragalus polysaccharide nanoparticles on septic cardiac dysfunction through inhibition of TLR4/NF-κB signaling pathway. Int J Biol Macromol. 2020; 153: 977-85.

20. Minneci P, Deans K, Natanson C, Eichacker PQ. Increasing the efficacy of anti-inflammatory agents used in the treatment of sepsis. Eur J Clin Microbiol Infect Dis. 2003; 22: 1-9.

21. Arndt P, Abraham E. Immunological therapy of sepsis: experimental therapies. Intensive Care Med. 2001; 27: 104-115.

22. van der Poll T. Immunotherapy of sepsis. Lancet Infect Dis. 2001; 1: 165-74.

23. Annane D, Sebille V, Charpentier C, Bollaert PE, Francois B, Korach J, et al. Effect of treatment with low doses of hydrocortisone and fludrocortisone on mortality in patients with septic shock. JAMA. 2002; 288: 862-71.

24. Annane D. Glucocorticoids in the treatment of severe sepsis and septic shock. Curr Opin Crit Care 2005; 11: 449-53.

25. Chen G, Xu Y. Biosynthesis of cerium oxide nanoparticles and their effect on lipopolysaccharide (LPS) induced sepsis mortality and associated hepatic dysfunction in male Sprague Dawley rats. Mater Sci Eng C. 2018; 83: 148-53.

26. Wang J, Wang H, Zhu R, Liu Q, Fei J, Wang S. Anti-infammatory activity of curcumin-loaded solid lipid nanoparticles in IL-1β transgenic mice subjected to the lipopolysaccharide-induced sepsis. Biomaterials. 2015; 53: 475-83.

27. Németh K, Leelahavanichkul A, Yuen PS, Mayer B, Parmelee A, Doi K, et al. Bone marrow stromal cells attenuate sepsis via prostaglandin E2-dependent reprogramming of host macrophages to increase their interleukin-10 production. Nat Med. 2009; 15: 42-9.
